# The evolution of anthropoid molar proportions

**DOI:** 10.1186/s12862-016-0673-5

**Published:** 2016-05-20

**Authors:** Katherine E. Carter, Steven Worthington

**Affiliations:** Department of Human Evolutionary Biology, Harvard University, 11 Divinity Avenue, Cambridge, 02138 USA; Institute for Quantitative Social Science, Harvard University, 1737 Cambridge Street, Cambridge, 02138 USA

**Keywords:** Molar proportions, Dental inhibitory cascade, Anthropoid primates, Evo-devo, Bayesian phylogenetic generalized linear mixed models

## Abstract

**Background:**

Developmental processes that underpin morphological variation have become a focus of interest when attempting to interpret macroevolutionary patterns. Recently, the Dental Inhibitory Cascade (dic) model has been suggested to explain much of the variation in mammalian molar size proportions. We tested the macroevolutionary implications of this model using anthropoid primate species (*n*=100), focusing on overall morphological patterns, as well as predictions made about molar size variability, direct developmental control, and diet.

**Results:**

Of the species sampled, 56 % had centroids that fell within regions of molar proportion morphospace consistent with the dic model. We also found that the third molar had greater variation in size than either the first or second molars, as expected by the model. Some dic model predictions were not supported, however, such as the expected proportion of *M*_2_/*M*_1_ when the third molar is absent. Furthermore, we found that some variability in third molar size could not be explained by the influence of the inhibitory cascade. Overall, we found considerable clade-specific differences in relative molar sizes among anthropoid primates, with hominoids and cercopithecins strongly divergent from dic model predictions, and platyrrhines, colobines, and papionins more consistent with the inhibitory cascade. Finally, we investigated reasons why some clades deviated from dic model expectations. Adaptations for frugivory (e.g., bunodont cusp relief) appeared to be one driver of relatively larger second molars and have evolved independently in multiple lineages of anthropoids.

**Conclusions:**

The dic model explains some of the variation in anthropoid primate molar proportions. However, there are interesting deviations away from this broad mammalian pattern, particularly in hominoids and cercopithecins, which suggest the model is only one of multiple mechanisms determining morphological variability in mammalian teeth.

**Electronic supplementary material:**

The online version of this article (doi:10.1186/s12862-016-0673-5) contains supplementary material, which is available to authorized users.

## Background

Mammalian dentition is complex and variable, adapting to the dietary, environmental, and social demands of a species’ niche. Despite the considerable range of variability in dental forms, certain morphological and evolutionary patterns emerge repeatedly in phylogenetically-distant taxa. For example, the hypocone (distolingual cusp on upper molars) evolved independently more than 20 times [1], hypsodonty (high-crowned molar teeth) evolved in multiple species of ungulates [2], lagomorphs [3], and South American rodents [4, 5], and tribosphenic molars may have evolved twice in mammals [6, 7] (but see [8]). During evolution, teeth are more frequently lost than gained [9–11] and the last developing teeth within a particular dental field are almost always lost first [12, 13]. While many of these patterns likely evolved in response to similar selective pressures [1, 14], there is evidence that developmental constraints limit molar shape [15]. Thus it is reasonable to assume that some of the dental variability of mammalian teeth is constrained by developmental processes.

In the past decade, several studies have explored developmental mechanisms potentially responsible for dental morphological variation [16–19]. Kavanagh and colleagues [16] developed the dental inhibitory cascade (DIC) model to explain the evolution and development of molar proportions. Through experimental alteration of developing mouse molars, the authors suggested that much of mammalian molar proportion diversity could be attributed to a simple, highly conserved pattern of differences in the timing and concentration of molecules that activate (*a*) or inhibit (*i*) molar initiation and proliferation. When the enamel knot of the earliest developing mandibular molar (*M*_1_) is initiated, it expresses inhibitory signals (including *ectodin, wise, Bmp3,* and *follistatin*) that inhibit, or greatly delay, the subsequent formation of *M*_2_ and *M*_3_. A change in the expression or timing of these inhibitory signals was proposed to determine the eventual size of *M*_2_ and *M*_3_, by altering the relative timing of molar initiation.

According to Kavanagh et al. [16], therefore, the patterning of molar sizes works as an inhibitory cascade, so that the timing of *M*_1_ development cumulatively affects the timing of *M*_2_ and *M*_3_ development, as the DIC mechanism operates along the developing tooth row. Decreased *M*_1_ inhibition allows *M*_2_ and *M*_3_ to form earlier and ultimately grow larger than *M*_1_, while increased inhibition restricts the size of subsequently developing molars and may eventually lead to their loss. The DIC model can be characterized by the following equation:

1 $$ y = 1 + \left[ (a - i) / i \right] \left(x - 1 \right),  $$

where *y* is molar area, as determined by position along the tooth row (*x*), and by the relative strength of activators (*a*) and inhibitors (*i*), defined by the *a*/*i* ratio. Molar areas are derived from Eq. 1 as *M*_1_=1,*M*_2_=*a*/*i*,*M*_3_=2(*a*/*i*)−1. Thus, balance between activation and inhibition leads to equal sized molars, while decreases in inhibition result in larger distal molar areas with a cumulative effect from *M*_2_ to *M*_3_. Since inhibition is hypothesized to cascade distally along the molar row, it is possible to predict the relative size of each molar using the following relationship:

2 $$ M_{3}/M_{1} = 2(M_{2}/M_{1}) - 1.  $$

As a test of the DIC model’s predictions in a macroevolutionary context, Kavanagh et al. [16] demonstrated that most of the molar patterns seen in murid rodents can be explained by alteration of the ratio of activation and inhibition molecules expressed during early tooth development.

One extension of the model is that in morphospace regions of high inhibition (*M*_1_≫*M*_2_≫*M*_3_), we would predict minimal temporal overlap between *M*_1_ and *M*_2_ calcification. As the level of inhibition decreases, however, the amount of overlap should increase, so that in morphospace regions with the lowest levels of inhibition (*M*_1_≪*M*_2_≪*M*_3_) temporal overlap would be substantial.

The DIC model highlights the importance of developmental timing in determining dental metrics, by establishing the pattern of hierarchical dependence within a particular developmental system and by asserting broad applicability across mammals (indicating stability under even the most extreme fluctuations in absolute developmental time).

While the model does not predict variation in cusp height, enamel thickness, or eruption schedule, one implication of the model is that molar size proportions are independent from these and other traits determined later in development. For example, our interpretation is that under this model, fruit bats, golden moles, and tree shrews not only exhibit similar molar size proportions, but proportions that were determined by fundamentally the same developmental interactions. The DIC model is innovative in suggesting that development may also drive molar size variation in addition to other variables, such as dietary adaptation, phylogenetic inertia, and allometry [20–22].

Researchers have begun to test the predictions of the DIC model in taxa, such as rodents and carnivores, which have shown rapid expansion of dental morphospace over their recent evolutionary history. A study of Mesozoic and Cenozoic mammals suggested that the DIC model could be plesiomorphic for this clade [23]. Yet, while some studies support the original findings of the DIC model [22, 24], others have presented evidence that calls into question its broad phylogenetic applicability [25–29]. For example, voles show an expansion of *M*_1_ size that drives molar proportions into morphospace (*M*_1_>*M*_2_<*M*_3_) previously thought unoccupiable [26], while canids exhibit a reduced major axis regression slope (0.45±0.07) between *M*_3_/*M*_1_ and *M*_2_/*M*_1_ that is much smaller than predicted by the DIC model [28]. Though the authors of both the above studies explain deviations from the DIC model as the result of changes in differential evolvability of the *M*_1_, the inability of the DIC model to explain all relationships between molar size proportions across Mammalia is worth further study. In particular, the model may not generalize to species with longer life histories, as the decay rates of activation and inhibition molecules may be sufficient enough to lessen the effect of the model [18, 30, 31].

Bernal et al. [29] assessed molar size proportion variation among platyrrhines, an extant radiation of South American primates, and found limited support for the DIC model. While the results of a phylogeny-dependent regression analysis corresponded with the predictions of the DIC model, an examination of platyrrhine molar proportions using ordinary least squares and reduced major axis regression, as well as an assessment of intraspecific variability in molar proportions, deviated from the DIC model’s predictions. The authors attribute these findings to the highly derived dentition of some platyrrhine clades, including the loss of the third molar in marmosets and tamarins, and reduction of the third molar in Cebinae [32].

We suggest that a broader consideration of the anthropoid dentition reveals multiple other morphological observations that are incongruent with the predictions of the DIC model. For example, great apes exhibit a *M*_1_<*M*_2_>*M*_3_ molar pattern, in contrast to the *M*_1_<*M*_2_<*M*_3_ pattern of cercopithecoids [33–35]. The *M*_1_<*M*_2_>*M*_3_ pattern is difficult to explain under the DIC model. Moreover, *M*_3_ agenesis in humans, which unlike in dwarfed marmosets and tamarins is polymorphic, has been reported to occur without large changes in relative *M*_2_ size [36–39].

Our aim in this paper is to test three predictions, and two extensions, of the DIC model using a broad sample of extant anthropoid primates. If anthropoid molar proportions follow the DIC model, this represents further evidence of the model’s robustness across mammalian taxa. If, however, anthropoids deviate from the DIC model’s predictions, it is possible that other factors play an equally important role in determining their relative molar sizes. Previous studies that have sought to test the DIC model’s developmental predictions have relied solely on measurements of molar crown size (e.g., [22, 29]). Anthropoid primates offer a unique opportunity to test the developmental predictions of the DIC model, as data are available on the timing of calcification in successive molars, allowing ontogenetic patterns to be evaluated. To test the model’s predictions we employ an integrative approach that considers dental metrics, development, diet, and function.

In the following section we report the results of testing three macroevolutionary predictions (1–3), and two extensions (4–5), of the DIC model:

1. *Molar area relationships:* The relationship among molar areas is: *M*_1_=1,*M*_2_=*a*/*i*,*M*_3_=2(*a*/*i*)−1. Balance between *a*/*i* leads to equal area molars, while increasing inhibition enlarges distal molar areas with a cumulative effect from *M*_2_ to *M*_3_. The relationship between *M*_3_/*M*_1_ and *M*_2_/*M*_1_ molar proportions can best be described by the linear equation: *M*_3_/*M*_1_=2(*M*_2_/*M*_1_)−1. Three regions of molar proportion morphospace are consistent with the dic model: a) a high *a*/*i* region where *M*_1_<*M*_2_<*M*_3_, b) a low *a*/*i* region where *M*_1_>*M*_2_>*M*_3_, and c) a region where *M*_3_/*M*_1_=2(*M*_2_/*M*_1_)−1. For mammals with three molars, *M*_2_ accounts for one-third of total lower molar area.
2. *Molar area variability:M*_3_ is predicted to have the greatest size variability of any lower molar.
3. *M*_3_ agenesis: *M*_3_ agenesis occurs when *M*_2_ is less than half the size of *M*_1_.
4. *Developmental timing:* In regions of molar proportion morphospace subject to high inhibition (*M*_1_≫*M*_2_≫*M*_3_) there is minimal temporal overlap between *M*_1_ and *M*_2_ calcification, while in morphospace regions of low inhibition (*M*_1_≪*M*_2_≪*M*_3_) temporal overlap is substantial.
5. *Primary dietary category and molar area proportions:* Molar proportions can be a measure of diet [16].

## Results and discussion

To test macroevolutionary predictions of the DIC model we used previously published lower molar occlusal area data from 100 extant anthropoid primates (Table 1; Additional file 1: Table S1; [40]). We analyzed these data using Bayesian phylogenetic generalized linear mixed models (PGLMM), and employed a Markov chain Monte Carlo (MCMC) approach to sample the posterior distribution of parameter space. Phylogenetic signal for each model was quantified using Pagel’s *λ* parameter [41]. All models achieved convergence to a stationary posterior distribution, as determined by Hiedelberger and Welch’s convergence diagnostic [42] and low levels (<0.1) of autocorrelation. Our MCMC sampling strategies resulted in effective sample sizes of 10,000 for each parameter. Detailed descriptions of the anthropoid sample, phylogenetic tree, morphometric variables, and regression models used are provided in the Methods section.

**Table 1:** Anthropoid species and specimen frequencies

Clade	Species *n*	Specimen *n*
Alouattinae	5	103
Atelinae	4	74
Callitrichini	6	178
Cebidae	5	195
Pitheciidae	5	102
Cercopithecini	18	626
Colobinae	21	693
Papionini	22	595
Hominidae	7	189
Hylobatidae	7	140
Total	100	2895

To evaluate if DIC model expectations were credible, given our anthropoid sample, we calculated 95 % highest density intervals (HDI) of posterior distribution parameter values. The DIC model specifies point predictions, rather than interval predictions, of parameter values. For any empirical dataset, the probability is therefore extremely small that molar proportions would exactly match DIC model expectations. To provide a more useful way of calculating the probability that DIC model predictions were correct, given our data, we created a *region of practical equivalence* (ROPE) around each DIC model parameter expectation. A ROPE is an interval that encloses values of the parameter that are, for practical purposes, negligibly different from the point value [43, 44]. We used the ROPE as a decision tool for determining whether DIC model predicted parameter values were credible and/or probable for the sampled anthropoid taxa.

To determine a range of slope/intercept values that might be deemed practically equivalent to the DIC mathematical model’s predictions, we used, as a starting point, experimental evidence reported in Kavanagh et al. [16]. This experimental evidence yielded a slope of 2.024 and intercept of -0.997, thus deviating slightly from the DIC mathematical model’s predictions of 2 and −1. For each PGLMM, we calculated posterior probabilities for ROPEs of several sizes. The inclusion of ROPEs of *any* size in our analyses was a conservative measure, increasing the chance of DIC model predictions being corroborated, compared with using only the point predictions of the strict mathematical model.

### Molar area corrected estimates

The product of maximum linear breadth and length measurements often overestimates molar occlusal area (Additional file 1: Figure S1). We employed a correction (see Methods for details) to make these rectangular areas more comparable to areas calculated by tracing outlines around molar occlusal perimeters. We found that the average difference between outline areas and corrected areas was much smaller (*oa*−*ca*: $\hat {\beta } = 0.079$, 95 % HDI −0.73, 0.99; in mm^2^) than that between outline areas and rectangular areas (*oa*−*ra*: $\hat {\beta } = 5.73$, 95 % HDI 4.87, 6.61). This indicated that corrected areas provided a better approximation to molar outline occlusal area (Additional file 1: Figure S2).

### Molar area relationships

#### Molar area proportions

The DIC model predicts that the relationship between *M*_3_/*M*_1_ and *M*_2_/*M*_1_ is best characterized by a line with a slope of 2 and an intercept of −1. In addition, two other regions of molar proportion morphospace are also consistent with the DIC model: a) a high *a*/*i* region where *M*_1_<*M*_2_<*M*_3_, and b) a low *a*/*i* region where *M*_1_>*M*_2_>*M*_3_. We calculated the posterior probability that anthropoid lower molar proportions can best be described by a line with parameters contained within ROPE intervals for the interspecific slope [1.90,2.10] and intercept [−1.10,−0.90]. We also determined whether these ROPEs contained credible parameter values for our anthropoid sample by estimating 95 % HDIs for the interspecific slope and intercept.

**Fig. 1:**
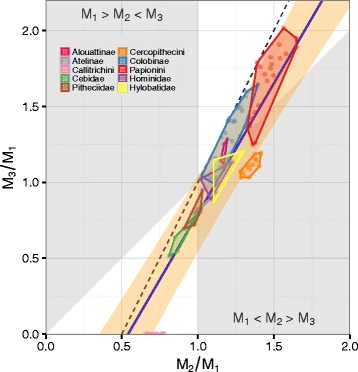
Anthropoid lower molar proportions (*n*=100). Points are species mean molar proportions. Convex hulls denote the range of species values for each higher ranked taxon. Solid line and surrounding polygon ribbon are the estimated mean and 95 % highest density interval for interspecific slope and intercept. Dashed line indicates DIC model’s predicted relationship between molar proportion ratios: *M*_3_/*M*_1_=2(*M*_2_/*M*_1_)−1. White regions are locations in molar proportion morphospace consistent with the DIC model: a high *a*/*i* region where *M*_1_<*M*_2_<*M*_3_ and a low *a*/*i* region where *M*_1_>*M*_2_>*M*_3_

We found that 56 % of the sampled anthropoid species’ centroids fell inside regions of molar proportion morphospace consistent with the DIC model (Fig. 1, Additional file 1: Figure S3). However, cercopithecins, hominids, and hylobatids occupied the *M*_1_<*M*_2_>*M*_3_ region of morphospace, indicating that their dental phenotypes cannot have originated from alterations in the *a*/*i* ratio. As in other mammalian clades [24, 26], we found that the *M*_1_>*M*_2_<*M*_3_ region of morphospace was unoccupied, suggesting that some dental morphologies result from developmental mechanisms that rarely occur in mammalian evolution.

**Fig. 2:**
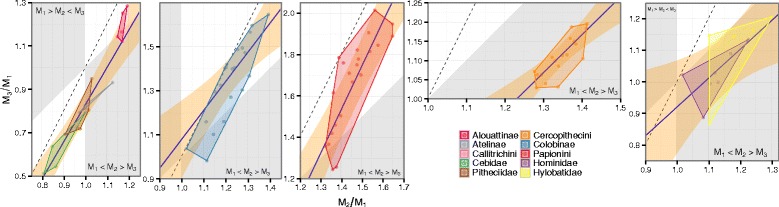
Lower molar proportions for clades within Anthropoidea. Points are species mean molar proportions. Convex hulls denote the range of species values for each higher ranked taxon. Solid line and surrounding polygon ribbon are the estimated mean and 95 % highest density interval for interspecific slope and intercept. Dashed line indicates DIC model’s predicted relationship between molar proportion ratios: *M*_3_/*M*_1_=2(*M*_2_/*M*_1_)−1. White regions are locations in molar proportion morphospace consistent with the DIC model: a high *a*/*i* region where *M*_1_<*M*_2_<*M*_3_ and a low *a*/*i* region where *M*_1_>*M*_2_>*M*_3_

For the entire anthropoid sample, we found that the posterior probability ($\mathbb {P}$) of the interspecific slope, $\mathbb {P}(\beta _{1\mathrm {B}} \in [1.90, 2.10]) = 0.10$, or intercept, $\mathbb {P}(\beta _{0} \in [-1.10, -0.90]) = 0.37$, being within the ROPE was low. The ROPE was partly encompassed by the 95 % HDI (1.42, 1.99), while the posterior mean was 1.72 (Table 2). For clades within Anthropoidea there was marked heterogeneity in slope and intercept estimates. Some taxa (e.g., platyrrhines and papionins) had posterior mean slope values closely mirroring DIC model predictions, while others (e.g., hominoids and cercopithecins) had much shallower slopes (Table 2, Fig. 2). Hominoids and cercopithecins both had 95 % HDIs that excluded the ROPE, indicating that DIC model predicted molar proportions are not credible for these taxa. All of the clades analyzed, however, exhibited low probabilities of slope or intercept values being within the DIC molar proportion ROPE (Additional file 1: Table S2, Figures S4–5).

**Table 2:** Relative molar area proportion PGLMM regression results for Anthropoidea and subclades

Clade/Model		Slope (Interspecific)					Intercept					*λ*		
		Mean	lhd	uhd	$\mathbb {P}$		Mean	lhd	uhd	$\mathbb {P}$		Mean	lhd	uhd
dic		2.00	1.90	2.10	1.000		−1.00	−0.90	−1.10	1.000				
Anthropoidea		1.72	1.42	1.99	0.102		−0.93	−1.32	−0.51	0.366		0.85	0.76	0.92
Platyrrhini		2.08	1.51	2.63	0.264		−1.30	−1.82	−0.77	0.156		0.71	0.37	0.91
Platyrrhini*		1.71	1.24	2.13	0.168		−0.88	−1.29	−0.42	0.352		0.34	<0.01	0.68
Catarrhini		1.63	1.28	1.96	0.056		−0.79	−1.24	−0.30	0.223		0.78	0.65	0.88
Hominoidea		0.91	0.17	1.63	0.003		0.01	−0.84	0.87	0.011		0.21	<0.01	0.73
Cercopithecoidea		1.73	1.36	2.11	0.162		−0.89	−1.39	−0.36	0.272		0.73	0.58	0.85
Colobinae		1.31	0.66	1.99	0.034		−0.23	−1.10	0.55	0.040		0.44	0.08	0.75
Cercopithecinae		2.04	1.56	2.51	0.329		−1.46	−2.14	−0.78	0.089		0.66	0.44	0.81
Cercopithecini		0.98	0.59	1.39	<0.001		−0.21	−0.76	0.32	0.006		0.09	<0.01	0.28
Papionini		2.11	1.52	2.77	0.243		−1.42	−2.34	−0.51	0.109		0.39	<0.01	0.74

^Mean; lhd; uhd^ posterior mean, lower and upper bound 95 % highest density levels

${\!\!~}^{\mathbb {P}}$posterior probability of parameter estimate being inside the rope: slope $\mathbb {P} \in [1.90, 2.10]$, intercept $\mathbb {P} \in [-0.90, -1.10]$

^*^non-callitrichin platyrrhines

These results indicate that anthropoids likely exhibit a shallower slope for molar proportion relationships than the DIC model predicts, suggesting that on average *M*_3_ increases in size less than twice as rapidly as *M*_2_. Just over one-half of sampled anthropoid species’ centroids fall within regions of molar proportion morphospace consistent with the DIC model, but several lower ranked clades (e.g., Hominidae, Hylobatidae, Cercopithecini) fall into the *M*_1_<*M*_2_>*M*_3_ region of morphospace and display molar proportion slopes that are substantially shallower than the DIC predicted slope. Catarrhines as a whole deviate more from the DIC model, and occupy regions of molar proportion morphospace with lower inhibition, than platyrrhines. Within the New World monkeys, only alouattines exhibited larger distal molars as a result of low inhibition. This variability highlights considerable clade-specific differences in molar proportion relationships among anthropoid primates. While the DIC model may provide a plausible explanation for molar proportions in platyrrhines, colobines, and papionins, the probability is low that the inhibitory cascade is a substantial factor driving relative molar size in hominoids and cercopithecins.

In the above analysis, we tested macroevolutionary predictions of the DIC model related to molar proportion morphospace. However, this analysis is potentially sensitive to the inclusion of ratios as variables in the regression model [45]. Therefore, as a robustness check, we tested two mathematically equivalent predictions of the DIC model that relate to the morphospace of raw molar areas, using regression models that included only raw molar area variables.

#### *M*_2_ relative area

For mammals with three molars, the DIC model predicts that *M*_2_ accounts for one-third of total lower molar area (*M*_T_). We defined a ROPE of [0.323, 0.343] around this one-third point value. For anthropoids, we found that the probability of relative *M*_2_ area being one-third of *M*_T_ was extremely low: $\mathbb {P}(M_{2}/M_{\mathrm {T}} \in [0.323, 0.343]) < 0.001$. The 95 % HDI (0.349,0.360) did not overlap the ROPE. This indicates that, for anthropoids, the proportion of lower molar total area attributable to *M*_2_ is likely larger than 0.333 (Table 3). Several lower ranked clades also followed this pattern of having relatively large *M*_2_s. Both hominoids (95 % HDI 0.347,0.384) and cercopithecins (95 % HDI 0.374,0.403) exhibited relatively larger *M*_2_s than anthropoids as a whole. In contrast, colobines (95 % HDI 0.327,0.362) and papionins (95 % HDI 0.328,0.356) deviated from this pattern, with 95 % HDIs that encompassed most of the ROPE (Additional file 1: Table S3, Figures S6–7). Overall, these results echo those of the molar proportions analysis. Hominoids and cercopithecins deviate most from DIC model predictions, while papionins, colobines, and platyrrhines are most consistent with the inhibitory cascade. Lucas et al. [46] have previously reported that *M*_2_ accounts for one-third of *M*_T_ area in anthropoids. In contrast, our results indicate that *M*_2_ is relatively larger in Anthropoidea, accounting for slightly more than one-third of *M*_T_ area, though we note the strong phylogenetic component driving variability in relative *M*_2_ size.

**Table 3:** *M*_2_ area as proportion of total lower molar area (*M*_T_), PGLMM regression results for Anthropoidea and subclades

Clade/Model		Slope (Interspecific)					Intercept					*λ*		
		Mean	lhd	uhd	$\mathbb {P}$		Mean	lhd	uhd	$\mathbb {P}$		Mean	lhd	uhd
dic		0.333	0.323	0.343	1.000		0.00	−0.10	0.10	1.000				
Anthropoidea		0.354	0.349	0.360	<0.001		0.25	−3.02	3.46	0.054		0.60	0.34	0.80
Platyrrhini		0.349	0.340	0.358	0.080		0.31	−0.25	0.85	0.125		0.18	<0.01	0.45
Platyrrhini ^∗^		0.351	0.340	0.363	0.061		0.08	−0.75	0.86	0.217		0.19	<0.01	0.49
Catarrhini		0.354	0.347	0.362	0.001		0.44	−2.95	3.96	0.047		0.61	0.38	0.77
Hominoidea		0.365	0.347	0.384	0.010		−1.86	−9.53	4.85	0.023		0.35	<0.01	0.68
Cercopithecoidea		0.342	0.334	0.349	0.602		1.37	−0.61	3.20	0.030		0.49	0.28	0.71
Colobinae		0.345	0.327	0.362	0.414		−0.41	−2.56	1.91	0.071		0.24	<0.01	0.52
Cercopithecinae		0.341	0.332	0.350	0.678		3.07	1.06	5.00	0.001		0.41	0.13	0.67
Cercopithecini		0.388	0.374	0.403	<0.001		0.04	−1.22	1.16	0.141		0.12	<0.01	0.29
Papionini		0.342	0.328	0.356	0.554		2.35	−1.03	6.00	0.018		0.36	<0.01	0.66

^Mean; lhd; uhd^ posterior mean, lower and upper bound 95 % highest density levels

${\!\!~}^{\mathbb {P}}$posterior probability of parameter estimate being inside the rope: slope $\mathbb {P} \in [0.323, 0.343]$, intercept $\mathbb {P} \in [-0.10, 0.10]$

^*^non-callitrichin platyrrhines

#### Molar areas

The DIC model predicts that the relationship among molar areas is: *M*_1_=1,*M*_2_=*a*/*i*,*M*_3_=2(*a*/*i*)−1. Equal area molars result when the *a*/*i* ratio is balanced, while decreasing inhibition results in larger distal molars with a cumulative effect from *M*_2_ to *M*_3_. Since the effect of relative *a*/*i* levels cascades distally along the molar field, one implication of this model is that the effect of *M*_1_ to *M*_2_ is repeated from *M*_2_ to *M*_3_.

**Fig. 3:**
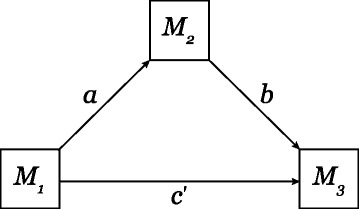
Path diagram of lower molar area relationships, illustrating the theoretical tripartite relationship between *M*_1_,*M*_2_ and *M*_3_ molar areas

We modeled the tripartite relationship of lower molar areas using path analysis to estimate how much the relationship between *M*_1_ and *M*_3_ area was mediated by *M*_2_ area. The ‘direct’ path (*c*^′^; Fig. 3) was the simple relationship between *M*_1_ and *M*_3_, while the ‘indirect’ path (*ab*) was the product of a proximal path (*a*) between *M*_1_ and *M*_2_ and a distal path (*b*) between *M*_2_ and *M*_3_ [47]. Our effect size measure was the proportion of total lower molar size variability accounted for by *M*_2_ acting as mediator: *pr*_*m*_=*ab*/*ab*+|*c*^′^|. Under a strict interpretation of the DIC model, *M*_1_ size influences *M*_3_ size only through the size of *M*_2_ (i.e., *pr*_*m*_=1). We defined a ROPE of [0.9, 1] for the DIC*pr*_*m*_ expectation.

For anthropoids, the probability was extremely low that *pr*_*m*_ was within the ROPE: $\mathbb {P}(\mathit {pr_{m}} \in [0.9, 1]) < 0.001$. The *pr*_*m*_ posterior mean (0.73) was just over two-thirds, with a 95 % HDI (0.68, 0.78) that did not encompass the ROPE (Table 4). There was some heterogeneity in *pr*_*m*_ among clades within Anthropoidea. Posterior means for all taxa fell well below DIC model predictions. The 95 % HDIs for platyrrhines and catarrhines excluded the ROPE, though some upper bound highest density limits approached 1 for catarrhine subclades. For cercopithecoids the *pr*_*m*_ was much higher than in other anthropoids ($\widehat {\mathit {pr_{m}}} = 0.86$, 95 % HDI: 0.75, 0.98) and partially overlapped the ROPE. Overall, the path analysis suggests, therefore, that Old World monkeys are more likely than other anthropoids to have molar sizes determined by an inhibitory cascade mechanism. However, even for cercopithecoids, the posterior probabilities of parameter estimates being inside the ROPE were moderate: $\mathbb {P}(\widehat {\mathit {pr_{m}}} \in [0.9, 1]) <= 0.42$ (Additional file 1: Table S4, Figure S8).

**Table 4:** Proportion of total variance mediated by *M*_2_ ($\widehat {\mathit {pr_{m}}}$), PGLMM regression results for Anthropoidea and subclades

Clade/Model		Mean	lhd	uhd	$\mathbb {P}$
dic		1.000	0.900	1.000	1.000
Anthropoidea		0.727	0.683	0.777	<0.001
Platyrrhini		0.657	0.595	0.726	<0.001
Platyrrhini*		0.656	0.578	0.746	0.003
Catarrhini		0.739	0.681	0.807	0.001
Hominoidea		0.621	0.142	1.000	0.141
Cercopithecoidea		0.855	0.752	0.984	0.233
Colobinae		0.819	0.608	1.000	0.274
Cercopithecinae		0.836	0.728	0.984	0.190
Cercopithecini		0.866	0.696	1.000	0.415
Papionini		0.837	0.703	1.000	0.262

^Mean; lhd; uhd^ posterior mean, lower and upper bound 95 % highest density levels

${\!\!~}^{\mathbb {P}}$posterior probability of parameter estimate being inside the rope: $\mathbb {P} \in \,[0.9, 1]$

^*^non-callitrichin platyrrhines

Only about two-thirds of the total effect of *M*_1_ size on *M*_3_ size was mediated through *M*_2_ size in anthropoids; much less than expected under the DIC model. While some of the inter-taxon heterogeneity in *pr*_*m*_ may be driven by body size (e.g., it is possible that constraints of the *a*/*i* model may be relaxed in larger-bodied taxa), both the largest and smallest taxa in our study seem to deviate most from the DIC model expectation of *pr*_*m*_. Surprisingly, those taxa for whom *M*_3_ is the largest lower molar (i.e., taxa hypothesized to have the least amount of inhibition) display the greatest mediation through *M*_2_ (e.g., compare Papionini to Platyrrhini). Overall, the path model either implies that there is a mechanism by which *M*_1_ size directly affects *M*_3_ size, or in some taxa a secondary developmental mechanism exists that solely affects *M*_2_ size.

Renvoise et al. [26] conducted mediation analysis on arvicoline rodents, finding that *M*_2_ size predicted *M*_3_ size (*b* path; Fig. 3) much more reliably than *M*_1_ size predicted *M*_3_ size (*c*^′^ path; Fig. 3). This is consistent with the DIC model, but Renvoise et al. did not calculate the overall effect of the indirect path through *M*_2_ (the product of *a* and *b* paths), which is a more appropriate summary of the overall mediation effect and a better comparator to the direct path (*c*^′^) [48]. It is therefore difficult to compare Renvoise et al.’s results with our own. However, both analyses suggest that while *M*_2_ size plays a large role in predicting *M*_3_ size, factors external to the DIC model are also involved in determining distal molar size.

### Body mass and fit/deviation from the DIC model

While not a prediction of the DIC model, the relationship between body size (as it influences absolute developmental timing) and fit/deviation from the DIC molar proportion line is of interest because long life histories (associated with larger body sizes) may provide a mechanism through which developmental constraints of the *a*/*i* pathway can be relaxed in a broad mammalian context.

We tested the association between species’ absolute perpendicular distance from the DIC molar proportion line and log body mass. For our entire sample of anthropoids, the 95 % HDI (−4.35,−0.39) for the specific-level slope was negative, indicating that increased deviation from the DIC model line is associated with a reduction in body size (Additional file 1: Figure S9a). Since callitrichins have extremely small body mass and deviate markedly from the DIC model line, we also fitted this model including only non-callitrichin anthropoids, to see if marmosets and tamarins were driving this negative relationship. We found that removing callitrichins reduced the likelihood of a negative association between body size and DIC model deviation, as the resulting 95 % HDI (−3.50,0.47) overlapped zero (Additional file 1: Figure S9b). In addition, among anthropoids, cercopithecins exhibit the greatest deviation from DIC model expectations and yet are relatively small-bodied. We therefore fitted a third model, including only non-cercopithecin and non-callitrichin anthropoids, to see how the small-bodied and extremely deviant cercopithecins were influencing the relationship between body mass and DIC deviation. We found that removing both of these clades resulted in a positive association between body size and DIC model deviation, as the posterior mean of the interspecific slope was positive (4.2), though the 95 % HDI (−0.70,9.04) overlapped zero (Additional file 1: Figure S9c).

### Molar area variability

The DIC model predicts that *M*_3_ will have the greatest size variability of any lower molar. We modeled the difference in average molar area variance (using the coefficient of variation for small sample sizes) between each lower molar type across the anthropoid sample. We found that *M*_3_ size was indeed more variable than either *M*_1_ size (posterior mean difference 0.015, 95 % HDI 0.009, 0.022) or *M*_2_ size (posterior mean difference 0.010, 95 % HDI 0.004, 0.017; *λ*=0.35, 95 % HDI 0.08, 0.63; Additional file 1: Figure S10). This is consistent with both the DIC model and previously theorized patterns of dental development in mammals (e.g., Butler’s [49] morphogenetic field theory).

### *M*_3_ agenesis

**Fig. 4:**
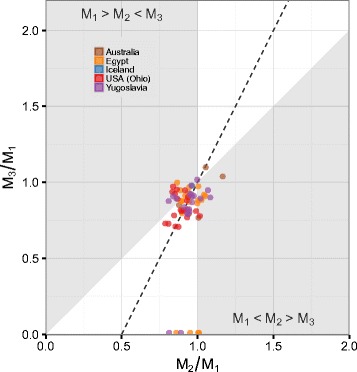
Lower molar proportions for modern humans polymorphic for *M*_3_ agenesis (*n*=66). Points are individual specimen molar proportions. Dashed line indicates DIC model’s predicted relationship between molar proportion ratios: *M*_3_/*M*_1_=2(*M*_2_/*M*_1_)−1. White regions are locations in molar proportion morphospace consistent with the DIC model: a high *a*/*i* region where *M*_1_<*M*_2_<*M*_3_ and a low *a*/*i* region where *M*_1_>*M*_2_>*M*_3_

The DIC model makes predictions about expected *M*_2_/*M*_1_ proportions when the third molar is absent. Specifically, *M*_3_ agenesis is predicted to occur when *M*_2_ is less than half the size of *M*_1_. In anthropoids, we instead found that *M*_3_ agenesis occurred when the *M*_2_/*M*_1_ ratio was much larger (posterior mean: 0.87, 95 % HDI: 0.64, 1.09; *λ*=0.82, 95 % HDI: 0.75, 0.89) and that the expected *M*_2_/*M*_1_ ratio under the DIC model had very low probability: $\mathbb {P}(M_{2}/M_{1} < 0.5 \mid M_{3}^{\mathit {ag}}) = 0.0003$. Within a sample of modern humans (*n*=66) polymorphic for *M*_3_ agenesis (Fig. 4, Additional file 1: Table S5), we also found that relative *M*_2_ size was much larger than predicted under the DIC model in individuals with congenitally missing *M*_3_s (posterior mean: 0.92, 95 % HDI: 0.83, 1.01) and that the DIC expected *M*_2_/*M*_1_ ratio was improbable: $\mathbb {P}(M_{2}/M_{1} < 0.5 \mid M_{3}^{\mathit {ag}}) = 0.0003$. In addition, the mean *M*_2_/*M*_1_ proportion for individuals not exhibiting *M*_3_ agenesis (posterior mean 0.95, 95 % HDI: 0.88, 1.03) was similar to those with congenitally missing *M*_3_s (posterior mean difference 0.03, 95 % HDI: −0.03, 0.09).

Third molar variations are seen with some frequency among primates. Modern humans, for example, are polymorphic in third molar number [50]. Evans et al. [51] have recently proposed that the DIC is a mechanism that explains both the relative and absolute sizes of permanent molars in modern humans, including the relative size of *M*_2_ when the third molar is absent. Our results demonstrate, in contrast, that modern humans have second molars subequal in size to first molars, whether or not the *M*_3_ is congenitally absent [52].

In marmosets and tamarins, *M*_3_ agenesis is likely to have occurred independently three times [32] and there is some reduction of *M*_2_ relative to other platyrrhines. However, our results agree with those of Bernal et al. [29] that *M*_2_ is still larger than predicted by the DIC model. One other platyrrhine, the extinct genus *Xenothrix*, has third molar agenesis, though it too has an *M*_2_/*M*_1_ proportion (0.74) much larger than the DIC model expectation [53].

The departure of callitrichins and modern humans from the expectations of the DIC developmental model suggests that mechanisms other than alterations in the *a*/*i* ratio may regulate variation in molar number and relative size. With the exception of certain syndromes, the developmental mechanisms leading to third molar agenesis are unknown, even in humans [18, 50]. There is some developmental evidence that supports a lack of association between relative *M*_2_ size and *M*_3_ agenesis, as Cai et al. [54] found that tooth size and tooth number were largely independent in rodents. Overall, anthropoids with third molar agenesis have *M*_2_/*M*_1_ proportions that are much larger than predicted by the DIC model, which contrasts with Carnivora, whose *M*_2_/*M*_1_ proportions range from larger (raccoons) to much smaller (Canidae) than expected under the model, with many forms being congruent with the model [24, 28].

### Developmental timing

**Fig. 5:**
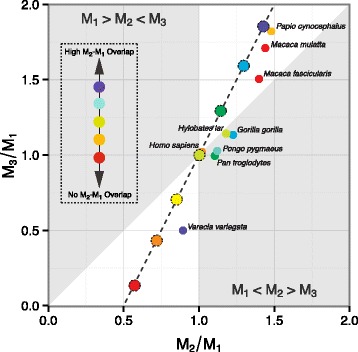
Developmental timing of lower molar calcification in primates (*n*=9). Points are species mean molar proportions. Colors denote degree of temporal overlap in *M*_2_– *M*_1_ calcification start times. Dashed line indicates DIC model’s predicted relationship between molar proportion ratios: *M*_3_/*M*_1_=2(*M*_2_/*M*_1_)−1. White regions are locations in molar proportion morphospace consistent with the DIC model: a high *a*/*i* region where *M*_1_<*M*_2_<*M*_3_ and a low *a*/*i* region where *M*_1_>*M*_2_>*M*_3_

One extension of the DIC model concerns the relative timing of molar development, specifically that temporal overlap in molar calcification initiation will increase with higher *a*/*i*. An assessment of nine primate species for which molar calcification data were available suggests the opposite trend. We predicted that temporal overlap would be highest in species with small *M*_1_s. Instead we found that genera with relatively large *M*_1_s, such as *Varecia*, had the greatest amount of temporal overlap between the development of *M*_1_ and *M*_2_, while genera like *Macaca*, with the smallest *M*_1_s, had the least amount of temporal overlap (Fig. 5). Though other factors, such as growth rates of subsequent molars, may also contribute to relative molar proportions, the evidence suggests that species with large posterior molars initiate them long after their smaller anterior molar crowns are complete.

A large *M*_3_ was associated with greater temporal separation between *M*_1_ and *M*_2_ initiation, even when controlling for *M*_1_ size. Though this pattern could also be consistent with a weak correlation between early stage (e.g., enamel knot formation and cusp differentiation) and late stage (e.g., calcification) developmental events, there is no histological evidence to support marked deviation in the relative schedule of these events. This suggests that, for anthropoids, the DIC model cannot fully explain variation in molar proportions.

### Primary dietary category and molar area proportions

**Fig. 6:**
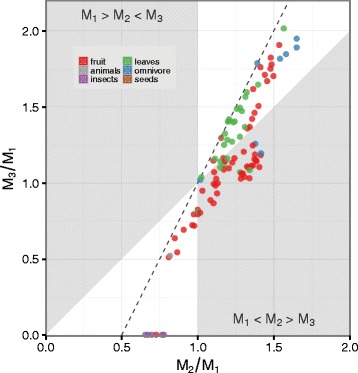
Anthropoid primary dietary category and lower molar proportions (*n*=100). Points are species mean molar proportions. Colors denote species’ primary dietary category. Dashed line indicates DIC model’s predicted relationship between molar proportion ratios: *M*_3_/*M*_1_=2(*M*_2_/*M*_1_)−1. White regions are locations in molar proportion morphospace consistent with the DIC model: a high *a*/*i* region where *M*_1_<*M*_2_<*M*_3_ and a low *a*/*i* region where *M*_1_>*M*_2_>*M*_3_

Kavanagh et al. [16] suggested that molar proportions could be associated with dietary preference. We tested this hypothesis by estimating whether anthropoid species that primarily eat fruit are more likely to occupy the *M*_1_<*M*_2_>*M*_3_ region of molar proportion morphospace than anthropoids that primarily eat other food items. We found that frugivorous anthropoids had much higher odds of occupying the *M*_1_<*M*_2_>*M*_3_ region of molar proportion morphospace (Odds Ratio: 9.51, 95 % HDI: 2.73, 32.3; *λ*=0.78, 95 % HDI: 0.68, 0.87; Fig. 6, Additional file 1: Figure S11). This equates to a 851 % (95 % HDI: 173 %, 3131 %) increase in the odds of an anthropoid species being frugivorous if *M*_2_ is its largest lower molar.

Four taxonomic groups (Hominidae, Hylobatidae, Cercopithecini, Atelinae) that predominantly occupy the *M*_1_<*M*_2_>*M*_3_ molar proportion morphospace have diets that are primarily frugivorous, with low-crowned bunodont molar cusps. Though some researchers have argued that molar proportions represent adaptations to processing food with different material properties [20, 55, 56], there is minimal evidence to support the *M*_1_<*M*_2_>*M*_3_ pattern as an adaptation for frugivory. There is, however, evidence to suggest that different cusp shapes and relief profiles have large effects on processing food with different mechanical properties [57, 58]. Broadly, folivores tend to have high-crowned or lophed teeth that aid the shearing of leafy plant material, while frugivores have lower, more bulbous, cusps that aid in mastication of soft pulpy fruit and the emission of juices [59]. If the strong selective pressure on reducing cusp height acts on genes that are involved in both the establishment of cusp relief and the regulation of molar sizes, then selection for lower cusp height would constrain the developmental pathways through which molars could evolve.

## Conclusions

Our results provide only limited support for the hypothesis that anthropoid molar proportions are partially governed by the dental inhibitory cascade model. We found a large amount of variability in relative and absolute molar sizes across anthropoid groups. Some clades (platyrrhines, colobines, and papionins) showed patterns of relative molar size consistent with changes in *a*/*i* concentrations, while others (hominoids and cercopithecins) diverged markedly from the expectations of the inhibitory cascade.

While the DIC model may have been the plesiomorphic model for mammalian molar proportions, in anthropoid primates and other taxa it is likely that secondary developmental pathways have influenced relative molar sizes [60]. We argue that the molar proportions of anthropoids result from the influence of two constraints in different directions: 1) co-option of the same signaling molecules (e.g., *Shh*, PDGF) for different developmental processes throughout dental evolution constrains the relationship between cusp height and relative molar proportion; and, 2) relaxation of constraints between activation and inhibition molecules caused by lengthening of absolute developmental time.

Co-option of genes occurs throughout dental evolution, including the development of toothed jaws [61, 62], regionalization in the dentition [63] and dental complexity [64, 65]. Dental features are interdependent, both within a tooth [66] and along a tooth row [67, 68], and it is likely that a similar relationship exists throughout developmental time between molar proportions and cusp relief. For example, *Shh* is known to regulate *Sostdc1* (a probable inhibitor of posterior molars) and alter the size of the enamel knot, influencing the ultimate size of cusps [17, 69, 70]. Similarly, altering concentrations of PDGF can change both molar size by 17 % and cusp height by 40 % [71]. Thus it is probable that selection for change in cusp relief has a related effect on molar proportions that drives species into regions of morphospace that are unexpected under the DIC model.

We propose that a relaxation of the constraints of the *a*/*i* pathway brought on by long life histories may allow the evolution of molar proportions that are inconsistent with the DIC model. Activation molecules are expressed only for a short period of time and these up-regulate inhibitory molecules. While larger-bodied individuals will typically have longer and more intense expression of both types of genes, there is an absolute limit on how long they can be expressed because of their molecular decay rate [72]. Smaller-bodied mammals that have slowed rates of embryonic development, such as callitrichins [73], may similarly be more prone to molar loss.

Thus, while small-bodied and quick developing species such as mice may be greatly constrained by *a*/*i* genes, larger-bodied taxa may evolve in ways that are not consistent with the DIC model. This possibility reiterates long-known concerns about using a small-bodied species with fast life history to generalize aspects of evo-devo across Mammalia [74]. Heterochronic change in the eruption timing of mammalian molars may also relax these developmental constraints, though this has not been tested in the developmental literature. This is potentially the case for many of the subfossil and living lemurids, who have a fast dental development schedule with early eruption of the molars [75], in addition to slow-developing callitrichins [29].

In this study, our aim was to investigate macroevolutionary predictions of the DIC model in anthropoid primates, specifically testing whether the developmental mechanisms proposed by the model matched observed morphological variation in relative molar areas. We found some congruence between the results of our analysis and the DIC model. Over one-half of the sampled species’ centroids fell within DIC model expected regions of molar proportion morphospace. In particular, platyrrhines, colobines, and papionins showed patterns of relative molar size consistent with changes in *a*/*i* concentrations. In anthropoids, however, it seems relatively easy to diverge from the strict predictions of the inhibitory cascade, which was particularly the case for hominoids and cercopithecins who occupied the *M*_1_<*M*_2_>*M*_1_ region of molar proportion morphospace. Diverse groups of non-primate taxa, for example canids [28], Notoungulata and Astrapotheria [22], and arvicoline rodents [26], also show divergence from DIC model expectations. Though we found only weak evidence of a positive association between deviation from the DIC model and body size, this could potentially be a result of the majority of the sampled anthropoid taxa having medium body size. A more robust test of this hypothesis will require a broader mammalian sample. While the DIC model explains some of the variation in mammalian molar proportions, there are likely other important developmental pathways being co-opted to produce molar rows with larger *M*_2_s than expected or those missing *M*_3_s. Further studies investigating the ontogenetic and molecular processes underlying different species will shed more light on the specific developmental pathways that determine molar proportions across mammals.

## Methods

### Materials

We obtained previously published data on lower molar areas of anthropoid primates from Plavcan [76]. This source reports maximum mesio-distal length and bucco-lingual breadth (separately for the trigonid and talonid) of each molar crown, to the nearest 1/100 mm, for 3283 specimens of 106 species. A total of 126 specimens were excluded from analysis due to either high levels of wear or absence of reporting wear condition. A further 24 specimens of indeterminate sex, 1 zoo specimen, and 221 specimens with incomplete lower molar dentitions were also excluded. To insure reasonably accurate estimates of species mean molar proportions and coefficients of variation, we included only species with at least four specimens that sampled both sexes. These exclusion criteria winnowed the original sample to 2,895 specimens from 100 taxa. Two subspecies of *Pan troglodytes* (*P. t. schweinfurthii* and *P. t. troglodytes*) were retained in the analysis. These anthropoid taxa represent a broad phylogenetic sample from Cercopithecoidea (*n*=61), Platyrrhini (*n*=25), and Hominoidea (*n*=14) (Table 1, Additional file 1: Table S1). We updated genus and species names to reflect the taxonomy of Wilson and Reeder [77].

For the analysis of *M*_2_/*M*_1_ proportions when *M*_3_ exhibits agenesis, we collected lower molar area data from 66 modern human specimens across 6 populations (Additional file 1: Table S5) from the Museum of Comparative Zoology, Harvard. To confirm *M*_3_ agenesis, radiographs were taken using a SKYSEA™ dental portable radiography machine and Ergonom self-developing X-ray film. Each quadrant was radiographed with the occlusal plane at *M*_2_ perpendicular to the X-ray. In addition, solely for the analysis of developmental timing, we obtained species mean molar proportion values of one strepsirrhine primate (*Varecia variegata*) from Swindler [59] (Additional file 1: Table S6).

To account for phylogenetic dependence among molar data during analysis, we used a majority-rule consensus tree of the 100 sampled anthropoid taxa, which was derived from the 10K Trees website and other sources (Additional file 1: Figure S12; [78, 79]). This phylogeny was generated from several sources of molecular data, including nuclear DNA, mitochondrial DNA, and Y-chromosome sequences. We used a single phylogenetic tree in our analyses, rather than a Bayesian posterior distribution of trees, as multiple trees were not available for a proportion of the sampled taxa. A potential limitation of the study, therefore, is that phylogenetic uncertainty was not accounted for in our models [80].

To examine the relationships between primate molar proportions and various traits of interest, we compiled data on: a) the calcification of successive molar crowns (temporal overlap of *M*_1_ and *M*_2_) from histological and radiographic sources (Additional file 1: Table S7), b) primary dietary category (fruit, leaves, insects, seeds, animals, omnivore) from the primatological literature (Additional file 1: Table S1), and c) body mass (Additional file 1: Table S1). Morphometric data, phylogenetic data (NEXUS format), and R code are archived in an online Zenodo data repository [40].

To test the efficacy of our molar area correction method, we collected molar measurements from three anthropoid species (*Alouatta seniculus, Cercopithecus mitis, Homo sapiens*) from the Museum of Comparative Zoology, Harvard. For each species, 10 wild-shot specimens (5 male, 5 female) were selected. Only adult specimens were used, as determined by complete fusion of spheno-occipital synchrondrosis and epiphyses on the clavicle and pelvis [81, 82].

### Data acquisition and preparation

We calculated molar crown areas for each anthropoid specimen using the product of bucco-lingual breadth (averaging trigonid and talonid bucco-lingual measurements) and mesio-distal length taken from Plavcan [76]. Since sample sizes for each sex were not always balanced, we calculated species-specific weighted means of molar crown areas by first aggregating to sex-specific species mean areas and then sex-pooled species mean areas (Additional file 1: Table S1). We also calculated a small-sample coefficient of variation (*cv*) for each molar type and species combination.

For the polymorphic modern human sample (*n*=66), we extracted outline areas from scaled superior occlusal photographs using the Polygon tool in Image J (v. 1.45r) [83]. Specimens were photographed using a Canon Digital Rebel XS 10 megapixel digital SLR camera fitted with an EF-S 60mm macro lens, following protocols established by Gómez-Robles and colleagues [84].

The product of linear breadth and length measurements often overestimates molar occlusal area (Additional file 1: Figure S1; [26]). We therefore used a correction to make these rectangular areas more comparable to areas calculated by tracing outlines around molar occlusal perimeters. Outline and rectangular areas from three species in different anthropoid clades, were extracted from scaled superior occlusal photographs using the Polygon and Straight-Segment tools in Image J (v. 1.45r) [83]. Specimens were photographed using the same equipment and protocols described above.

We calculated areas from length (*l*) and breadth (*b*) variables to estimate each molar’s area as both a rectangle (*ra*=*bl*) and ellipse (*ea*=*π*(*bl*)/4) and then compared these estimates with the molar’s outline area (*oa*) to solve for a coefficient (*x*) of molar shape: *x*=*oa*−*ea*/*ra*−*ea*. We then created corrected areas (*ca*) by applying these coefficients, solving for area: *ca*=*ra*(*x*)+*ea*(1−*x*). We tested the usefulness of our correction method by comparing the difference between outline areas and corrected areas to the difference between outline areas and rectangular areas using a GLMM.

### Selection of methods

Several studies investigating DIC model predictions with mammalian molar areas have used a reduced (standardized) major axis estimator (RMA; e.g., [16, 22, 26, 28, 29]), which assumes both *Y* and *X* are random variables measured with error and thus seeks to minimize error orthogonal to the model. We chose not to use RMA, since this method is known to produce large over-estimates of slope when the ratio of error variances between *Y* and *X* is not unity [85, 86] or when the ratio of error variances does not equal the ratio of variances [87]. With empirical data, both these assumptions are unlikely to be met [88]. In addition, species-level observations are correlated with phylogeny [89] and therefore violate the assumption of statistical independence that is fundamental to many regression models, including RMA. Accounting for phylogenetic dependence in the data is possible, but complicated in a RMA framework. Currently the only software implementation is for the simple case of bivariate Gaussian data. Notably, none of the above studies accounted for phylogenetic dependence in their RMA models.

One alternative regression method some studies have used (e.g., [29]) is based on a generalized least squares estimator (PGLS) with an additional parameter (*λ*) that scales off-diagonal elements of the variance-covariance matrix in the error term, according to an hypothesis of phylogenetic relationships. This method, in common with the ordinary least squares estimator, treats explanatory variables as fixed and therefore minimizes error only in the *Y* variable. In addition, it is currently not possible to fit models that incorporate individual-level data, and thus measurement error, using PGLS.

Due to the shortcomings of the above methods, we elected to use Bayesian phylogenetic generalized linear mixed models, estimated using a Markov chain Monte Carlo approach. It is straightforward to incorporate phylogenetic information into PGLMMs to account for interspecies dependence [90]. In addition, the PGLMM estimator has the advantage of being able to model individual-level data, thus allowing intraspecific variance (both polymorphism and measurement error) to be accounted for in estimates of among-species relationships [80, 91]. Variation below the species level is considered a source of uncertainty (error) in specific-level variables and, if ignored, can produce biased estimates of among-species relationships and inflated type I error rates [92–96]. Incorporating specific-level error in models often decreases the precision with which parameters can be estimated, but provides more realistic interval estimates for those parameters. We fitted all models using the MCMCglmm() function from package MCMCglmm v. 2.21 [91] in R v. 3.1.3 [97].

### Phylogenetic generalized linear mixed models

#### General model components and priors

The basic components of the Bayesian PGLMM are:

3a $$\begin{array}{*{20}l} \mathbf{y} &\sim \mathcal{D} \left(\mu, \phi \right),  \end{array} $$

3b $$\begin{array}{*{20}l} \mu &= \mathrm{g}^{-1} \left(\mathbf{l} \right),  \end{array} $$

3c $$\begin{array}{*{20}l} \mathbf{l} &= \boldsymbol{\eta} + \mathbf{e},  \end{array} $$

3d $$\begin{array}{*{20}l} \boldsymbol{\eta} &= \mathbf{W}\boldsymbol{\theta}.  \end{array} $$

The random component (Eq. 3a) comprises a vector of observed trait values **y** and a probability distribution $\mathcal {D}$ with mean *μ* and variance *ϕ*. For the logistic distribution *ϕ* is a constant (*ϕ*=*π*^2^/3), while for the Gaussian distribution it has to be estimated (*ϕ*=*σ*^2^). The systematic component (Eq. 3d) contains the linear part of the model, with the linear predictor ***η*** composed of a design matrix **W** that relates the explanatory variables (**w**_1_…**w**_*n*_) to the data and a vector of location effects ***θ*** (‘fixed’ and ‘random’ effects). In between these two parts, a hierarchical layer is added to the model (Eq. 3c), where **l** is a hypothetical latent variable composed of the linear predictor ***η*** and a vector of residuals **e** (or the effect due to additive dispersion for non-Gaussian models). The final component (Eq. 3b) joins the random and systematic parts of the model together using a link function g(·), or more usually its inverse g^−1^(·). This function is used to relate the latent variable **l** to the observed data **y**, by transforming **l** into a quantity g^−1^(**l**) that is the expectation of the distribution of **y**. For the Bernoulli distribution this would be the logistic function *μ*=logit^−1^(**l**)=logistic(**l**) while for the Gaussian distribution it is the identity function *μ*=**l**.

In our analyses, when the response variable was Gaussian and an identity link function specified, then **y** was assumed to be normally distributed ($\mathcal {N}$) with expectation equal to the latent variable:

4a $$ \mathbf{y} \sim \mathcal{N} \left(\mathbf{l} \right).  $$

When the response variable was binary and a logit link function specified, then **y** was assumed to be Bernoulli distributed ($\mathcal {B}$) with expectation equal to the logistically transformed latent variable:

4b $$ \mathbf{y} \sim \mathcal{B} \left(\text{logistic} \left(\mathbf{l} \right) \right).  $$

In a Bayesian analysis there is no distinction between fixed and random effects, with all effects treated as random [98]. However, since these terms are prevalent in the literature we note that **W** can be further deconstructed into design matrices for ‘fixed’ (**X**) and ‘random’ (**Z**) effects, and ***θ*** can be deconstructed into vectors of ‘fixed’ (***β***) and ‘random’ (***γ***) effects parameters:

5a $$\begin{array}{*{20}l} \mathbf{W} &= \left[ \mathbf{X}, \mathbf{Z} \right] \end{array} $$

5b $$\begin{array}{*{20}l} \boldsymbol{\theta} &= \left[ \boldsymbol{\beta}, \boldsymbol{\gamma} \right]. \end{array} $$

The ‘fixed’ effects were assumed a priori to be independently distributed with zero mean and *specified* variance (${\sigma ^{2}_{b}}$) or scale (*γ*_*b*_):

6a $$\begin{array}{*{20}l} \boldsymbol{\beta} &\sim \mathcal{N} \left({0}, {\sigma^{2}_{b}} \mathbf{I} \right)  \end{array} $$

6b $$\begin{array}{*{20}l} \boldsymbol{\beta} &\sim \mathcal{C} \left({0}, \gamma_{b} \mathbf{I}\vphantom{{\sigma^{2}_{b}}} \right),  \end{array} $$

where **I** is an identity matrix. To represent diffuse prior knowledge of the ‘fixed’ effects, for Gaussian response models (Formula 6a) we used a normal distribution and set ${\sigma ^{2}_{b}}$ to be 1×10^8^, while for categorical responses (Formula 6b) we used a Cauchy distribution ($\mathcal {C}$) with *γ*_*b*_ equal to *π*^2^/3+*v*, where *v* is the total variance of the ‘random’ and residual effects. This Cauchy prior is approximately flat on the probability scale [99].

In our models, the ‘random’ effects design matrix was composed of:

7 $$ \mathbf{Z}\boldsymbol{\gamma} = \mathbf{p}\gamma_{1} + \mathbf{s}\gamma_{2},  $$

where **p** is a phylogenetic (correlated) random variable, **s** is a species-specific (i.i.d.) random variable, and *γ*_*i*_ are vectors of ‘random’ effects parameters. The above terms accounted for between-species variation in intercepts due to both phylogenetic and multiple measurement effects [100]. It is also possible to model among-species variation in slopes, but this requires a large intra-specific sample size for all species [101]. Since interspecies slope heterogeneity in our sample of anthropoids was low (Additional file 1: Figure S13), and sample sizes for some species were small (Additional file 1: Table S1), we did not increase the complexity of our models by adding terms for random slopes.

The ‘random’ effects were also assumed to be normally distributed about zero, but with variances (${\sigma ^{2}_{p}}$ and ${\sigma ^{2}_{s}}$) inferred *a posteriori*:

8a $$\begin{array}{*{20}l} \mathbf{p} &\sim \mathcal{N} \left(0, {\sigma^{2}_{p}} \boldsymbol{\Sigma} \right) \end{array} $$

8b $$\begin{array}{*{20}l} \mathbf{s} &\sim \mathcal{N} \left(0, {\sigma^{2}_{s}} \mathbf{I}^{\vphantom{{\sigma^{2}_{p}} \boldsymbol{\Sigma}}} \right), \end{array} $$

where ***Σ*** is a scaled matrix of phylogenetic covariances calculated from an ultrametric majority-rule consensus tree of the sampled anthropoid taxa [80]. Including the **p** term in the model thus makes the assumption that phylogenetic effects are correlated according to ***Σ***. To embody our defuse prior knowledge of the ‘random’ effects we specified parameter expanded priors for **p** and **s**, with scale = 1, degree of belief = 1, mean = 0, and variance =1×10^3^ [91]. It has been suggested that parameter expanded priors are preferable when weakly informative priors are sought [102].

The error term (**e**) was also assumed to be normally distributed:

9 $$ \mathbf{e} \sim \mathcal{N} \left(0, {\sigma^{2}_{e}} \mathbf{I} \right),  $$

where the residual variance ${\sigma ^{2}_{e}}$ was estimated for Gaussian responses and fixed at *π*^2^/3 for Bernoulli responses.

#### Phylogenetic signal

Phylogenetic signal for each model was calculated using Pagel’s [41] *λ* parameter:

10 $$ \lambda = \frac{{\sigma^{2}_{p}}}{{\sigma^{2}_{p}} + {\sigma^{2}_{s}} + {\sigma^{2}_{e}}},  $$

where ${\sigma ^{2}_{e}}$ was estimated for Gaussian responses and fixed at *π*^2^/3 for categorical responses. The *λ* parameter is measured on the interval [0, 1] and multiplies off-diagonal elements of ***Σ*** to reflect the pattern of observed covariance in trait values under a Brownian motion model.

#### Within-group centering for species-level effects

When multiple measurements for each species are included in regression models the resulting ***β*** parameters measure a combination of between- and within-species effects. To disentangle inter- from intra-specific effects we therefore used within-group centering [100, 103]. This method partitioned each predictor **x** into two components, one containing the specific mean of **x**, the other containing a measure of within-species variability (deviation of individual measurements from the specific mean). For individual *j* belonging to species *i* the generic model was thus:

11a $$ y_{ij} = \beta_{0} \texttt{1} + \beta_{\mathrm{B}} \bar{x}_{i} + \beta_{\mathrm{W}} \left[ x_{ij} - \bar{x}_{i} \right] + p_{i} + s_{i} + e_{ij},  $$

where:

11b $$ \bar{x}_{i} = \frac{1}{J_{i}} \sum\limits_{j=1}^{J_{i}} x_{ij},  $$

with *J*_*i*_ being the number of individuals in species *i*. For each predictor **x**, there were thus two slopes: *β*_B_ the between-species slope and *β*_W_ the (common) slope within each species [100, 103].

#### MCMC sampling and model convergence criteria

For Gaussian response models we sampled from the posterior distribution using MCMC for 1.1×10^7^ iterations, with a burn-in period of 1×10^6^ and thinning interval of 1000. For Bernoulli response models the posterior MCMC sample was run for 1.5×10^7^ iterations, with a burn-in period of 3×10^6^ and thinning interval of 1200. We determined that models had converged to a stationary posterior distribution when Hiedelberger and Welch’s diagnostic test [42] was passed and autocorrelation levels dipped below 0.1.

### Data analysis

#### Molar area proportion relationships

To test the first prediction of the DIC model we fitted a regression model of molar proportions. The relationship between *M*_3_/*M*_1_ and *M*_2_/*M*_1_ occlusal area proportions can be described by the following model:

12 $$ \begin{aligned} \frac{M_{3\mathit{ij}}}{M_{1\mathit{ij}}} = \beta_{0} \texttt{1} &+ \beta_{1\mathrm{B}} \frac{\overline{M}_{2i}}{\overline{M}_{1i}} + \beta_{1\mathrm{W}} \left[ \frac{M_{2\mathit{ij}} - \overline{M}_{2i}}{M_{1\mathit{ij}} - \overline{M}_{1i}} \right] \\ & + \gamma_{1} p_{i} + \gamma_{2} s_{i} + e_{\mathit{ij}}, \end{aligned}  $$

where the parameters *β*_0_ and *β*_1B_ are the between-species intercept and slope of a line representing the population average of *M*_3_/*M*_1_ conditional on a given value of *M*_2_/*M*_1_, and *β*_1W_ is the (pooled) within-species slope.

#### *M*_2_ relative area

To determine if *M*_2_ area accounts for one-third of lower molar total area, we regressed the sum of lower molar areas on *M*_2_ area using the following model:

13 $$ \begin{aligned} M_{2\mathit{ij}} = \beta_{0} \texttt{1} &+ \beta_{1\mathrm{B}} \overline{M}_{\mathrm{T}i} + \beta_{1\mathrm{W}} \left[ M_{\mathrm{T}\mathit{ij}} - \overline{M}_{\mathrm{T}i} \right] \\ & + \gamma_{1} p_{i} + \gamma_{2} s_{i} + e_{\mathit{ij}}, \end{aligned}  $$

where *M*_2_ is the occlusal surface area of the second lower molar, *M*_T_ is the sum of all three lower molar areas, and *β*_1B_ and *β*_1W_ are between- and (pooled) within-species estimates of the proportion of total lower molar area accounted for by *M*_2_.

#### Molar area relationships

We assessed how much the relationship between *M*_1_ and *M*_3_ area was mediated by *M*_2_ area, using two models (Eqs. 14, b) to estimate parameters of the three pathways representing molar area relationships:

14a $$\begin{array}{*{20}l} M_{3\mathit{ij}} &= \beta_{0} \texttt{1} + \beta_{1\mathrm{B}} \overline{M}_{1i} + \beta_{1\mathrm{W}} \left[ M_{1\mathit{ij}} - \overline{M}_{1i} \right] \\ &\hphantom{{}=\beta_{0} \texttt{1}} + \beta_{2\mathrm{B}} \overline{M}_{2i} + \beta_{2\mathrm{W}} \left[ M_{2\mathit{ij}} - \overline{M}_{2i} \right] \\ &\hphantom{{}=\beta_{0} \texttt{1}} + \gamma_{1} p_{i} + \gamma_{2} s_{i} + e_{\mathit{ij}},  \end{array} $$

14b $$\begin{array}{*{20}l} M_{2\mathit{ij}} &= \beta_{0}' \texttt{1} + \beta_{1\mathrm{B}}' \overline{M}_{1i} + \beta_{1\mathrm{W}}' \left[ M_{1\mathit{ij}} - \overline{M}_{1i} \right] \\ &\hphantom{{}=\beta_{0} \texttt{1}} + \gamma_{1} p_{i} + \gamma_{2} s_{i} + e_{\mathit{ij}}, \end{array} $$

14c $$\begin{array}{*{20}l} \widehat{ab} &= \frac{1}{T} \sum\limits_{t=1}^{T} \, \beta_{2\mathrm{B}}^{(t)}\beta_{1\mathrm{B}}^{\prime(t)},  \end{array} $$

14d $$\begin{array}{*{20}l} \lvert \widehat{c^{\prime}} \rvert &= \frac{1}{T} \sum\limits_{t=1}^{T} \, \lvert \beta_{1\mathrm{B}}^{(t)} \rvert,  \end{array} $$

14e $$\begin{array}{*{20}l} \widehat{\mathit{pr_{m}}} &= \frac{\widehat{ab} }{ \widehat{ab} + \lvert \widehat{c^{\prime}} \rvert}, \end{array} $$

where *M*_*i*_ is the occlusal surface area and *β*_*i*B_ and *β*_*i*W_ are the between- and (pooled) within-species slopes of the *i*^th^ lower molar. The indirect effect was calculated using Eq. 14c, where $\beta _{2\mathrm {B}}^{(t)}$ and $\beta _{1\mathrm {B}}^{\prime (t)}$ are the *t*^th^ parameter estimates for *t*=1,…,*T*MCMC draws from the posterior distribution. The direct effect was calculated in the same manner using Eq. 14d. Point estimates of the mediated ($\widehat {ab}$) and direct ($\lvert \widehat {c^{\prime }} \rvert $) effects are the mean of these individual draws, while the 95 % HDI is given by the (.025,.975) sample quantiles of the posterior draws [48]. We calculated the proportion mediated $\widehat {\mathit {pr_{m}}}$ as the ratio of indirect effect to total effect, with the latter being the sum of the indirect effect and the absolute value of the direct effect (Eq. 14e) [48, 104].

#### Body mass and fit/deviation from the DIC model

We used the following model to test the association between fit/deviation from the DIC model line and body mass:

15 $$ \begin{aligned} \text{ln}(\texttt{mass}_{i}^{\text{kg}}) = \beta_{0} \texttt{1} &+ \beta_{1\mathrm{B}} \lvert \texttt{dic}_{i}^{\texttt{dev}} \rvert \\ & + \beta_{1\mathrm{W}} \left[ \lvert \texttt{dic}_{\mathit{ij}}^{\texttt{dev}} \rvert - \lvert \overline{\texttt{dic}}_{i}^{\texttt{dev}} \rvert \right] \\ & + \gamma_{1} p_{i} + \gamma_{2} s_{i} + e_{\mathit{ij}}, \end{aligned}  $$

where $\text {ln}(\texttt {mass}_{i}^{\text {kg}})$ is the natural log of species mean body mass and $\lvert \texttt {dic}_{\mathit {ij}}^{\texttt {dev}} \rvert $ is the absolute perpendicular distance from the DIC model line to each individual’s location in molar proportion space.

#### Molar area variability

We tested whether average third molar area variability ($M_{3}^{\mathit {cv}}$) across all anthropoid species sampled was larger than either average first ($M_{1}^{\mathit {cv}}$) or second ($M_{2}^{\mathit {cv}}$) molar area variability using the following model:

16a $$ \widehat{\mathit{cv}}_{i} = \beta_{0} \texttt{1} + \beta_{1} M_{1i} + \beta_{1} M_{2i} + \gamma_{1} p_{i} + \gamma_{2} s_{i} + e_{\mathit{i}},  $$

where:

16b $$ M_{1i} =\left\{ \begin{array}{ll} 1 & \text{if}~ \widehat{\mathit{cv}}_{i} ~\mathrm{is~ from}~ M_{1} \\ 0 & \text{if}~ \widehat{\mathit{cv}}_{i}~ \mathrm{is~ not~ from~} M_{1} \end{array} \right.  $$

and

16c $$ M_{2i} =\left\{ \begin{array}{ll} 1 & \text{if}~ \widehat{\mathit{cv}}_{i}~ \mathrm{is~ from}~ M_{2} \\ 0 & \text{if}~ \widehat{\mathit{cv}}_{i}~ \mathrm{is~ not~ from}~ M_{2} \end{array} \right.  $$

and where $\widehat {\mathit {cv}}$ is the sample estimate of the coefficient of variation for small sample sizes:

16d $$ \widehat{\mathit{cv}} = \left(1 + \frac{1}{4n} \right) \left(\frac{s}{\bar{x}} \right).  $$

We used $M_{3}^{\mathit {cv}}$ as the reference level for the indicator variables.

#### *M*_3_ agenesis

We fitted the following model to determine whether *M*_3_ agenesis across our anthropoid sample is associated with values of *M*_2_/*M*_1_<0.5:

17a $$ \frac{\overline{M}_{2\mathit{ij}}}{\overline{M}_{1\mathit{ij}}} = \beta_{1} M_{3\mathit{i}}^{\mathit{ag}} + \beta_{2} M_{3\mathit{i}}^{\mathit{re}} + \gamma_{1} p_{i} + \gamma_{2} s_{i} + e_{\mathit{ij}},  $$

where:

17b $$ M_{3\mathit{i}}^{\mathit{ag}} =\left\{ \begin{array}{ll} 1 & \mathrm{if~species}~ \textit{i}~ \text{exhibits}~ M_{3}~ \text{agenesis} \\ 0 & \mathrm{if~ species}~ \textit{i}~\text{retains}~ M_{3} \end{array} \right.  $$

and

17c $$ M_{3\mathit{i}}^{\mathit{re}} =\left\{ \begin{array}{ll} 1 & \mathrm{if~ species}~ \textit{i}~ \text{retains}~ M_{3} \\ 0 & \mathrm{if~ species}~ \textit{i}~ \text{exhibits}~ M_{3}~ \text{agenesis}. \end{array} \right.  $$

We also fitted a model to determine whether *M*_3_ agenesis within a polymorphic modern human sample is associated with values of *M*_2_/*M*_1_<0.5:

18a $$ \frac{\overline{M}_{2\mathit{ij}}}{\overline{M}_{1\mathit{ij}}} = \beta_{1} M_{3\mathit{ij}}^{\mathit{ag}} + \beta_{2} M_{3\mathit{ij}}^{\mathit{re}} + \gamma_{1} r_{i} + e_{\mathit{ij}},  $$

where **r** is a population-specific (i.i.d.) random variable with *i* levels, distributed as:

18b $$ \mathbf{r} \sim \mathcal{N} \left(0, {\sigma^{2}_{s}} \mathbf{I} \right),  $$

and where:

18c $$ M_{3\mathit{ij}}^{\mathit{ag}} =\left\{ \begin{array}{ll} 1 & \mathrm{if~individual}~ \textit{j}~ \text{exhibits}~ M_{3}~ \text{agenesis} \\ 0 & \mathrm{if~individual}~ \textit{j}~ \text{retains}~ M_{3} \end{array} \right.  $$

and

18d $$ M_{3\mathit{ij}}^{\mathit{re}} =\left\{ \begin{array}{ll} 1 & \mathrm{if~individual}~ \textit{j}~ \text{retains}~ M_{3} \\ 0 & \mathrm{if~ individual}~ \textit{j}~ \text{exhibits}~ M_{3}~ \text{agenesis}. \end{array} \right.  $$

#### Primary dietary category and molar area proportions

We used the following logistic regression model to test the association between primary dietary category and the relative size of *M*_2_:

19a $$ \begin{aligned} \mathbb{P} & \bigl(M_{1\mathit{ij}} < M_{2\mathit{ij}} > M_{3\mathit{ij}} = 1 \mid \mathbf{W}\boldsymbol{\theta}, \mathbf{e} \bigr) \\ & = \text{logistic} \bigl(\beta_{0} \texttt{1} + \beta_{1} \texttt{fruit}_{i} + \gamma_{1} p_{i} + \gamma_{2} s_{i} + e_{i} \bigr), \end{aligned}  $$

where:

19b $$ \texttt{fruit}_{i} =\left\{ \begin{array}{ll} 1 & \mathrm{if~ species}~ \textit{i}~ \mathrm{is~ a~ frugivore} \\ 0 & \mathrm{if~ species}~ \textit{i}~ \mathrm{is~ not~ a~ frugivore}. \end{array} \right.  $$

This equation models the probability of being in the *M*_1_<*M*_2_>*M*_3_ region of molar proportion morphospace as a function of a species’ primary dietary category.

## Ethics and consent to participate

Not applicable.

## Consent to publish

Not applicable.

## Availability of data and materials

The morphometric and phylogenetic data supporting the results of this article, together with an R script that replicates the analyses, are available in the Zenodo repository (http://dx.doi.org/10.5281/zenodo.50108).

## Additional file

Additional file 1
**Table S1.** Anthropoid (*n*=100) lower molar proportions, body mass, and diet summary data. **Table S2.** Molar area proportion pglmm posterior probabilities. **Table S3.** Relative *M*_2_ area pglmm posterior probabilities. **Table S4.** Proportion mediated (*p* *r*_*m*_) pglmm posterior probabilities. **Table S5.** Modern human (*n*=66) lower molar proportions data. **Table S6.** Strepsirrhine (*n*=1) lower molar proportions summary data. **Table S7.** Primate molar crown formation time data. **Figure S1.** Molar area correction coefficients method. **Figure S2.** Molar area correction coefficients test. **Figure S3.** Anthropoid lower molar proportions of individual specimens (*n*=2,895). **Figure S4.** Posterior density of molar proportion slope (interspecific). **Figure S5.** Posterior density of molar proportion intercept. **Figure S6.** Posterior density of relative *M*_2_ area slope (interspecific). **Figure S7.** Posterior density of relative *M*_2_ area intercept. **Figure S8.** Posterior density of proportion mediated (interspecific). **Figure S9.** Ln body mass as a function of fit/deviation from the dic molar proportion model line. **Figure S10.** Anthropoid lower molar proportions intra-specific variation. **Figure S11.** Anthropoid lower molar proportions with respect to relative *M*_2_ area. **Figure S12.** Molecular phylogeny of anthropoid primates (*n*=100). **Figure S13.** Anthropoid lower molar proportions intra-specific error. **Supplementary references.** (1–43). (PDF 3891 kb).

## Abbreviations

*c**v*: coefficient of variation for small sample sizes
dic: dental inhibitory cascade
hdi: highest density interval
*M*_1_: first lower molar
*M*_2_: second lower molar
*M*_3_: third lower molar
$\mathbb {P}$: posterior probability
rope: region of practical equivalence
