# Supplementary material for: The evolution of anthropoid molar proportions
Source: BMC Evol Biol. 2016 May 20;16:110. doi: 10.1186/s12862-016-0673-5 (PMC4875740; doi:10.1186/s12862-016-0673-5)
Supplement: Additional file 1 — Table S1. Anthropoid (n=100) lower molar proportions, body mass, and diet summary data. Table S2. Molar area proportion pglmm posterior probabilities. Table S3. Relative M 2 area pglmm posterior probabilities. Table S4. Proportion mediated (p r m) pglmm posterior probabilities. Table S5. Modern human (n=66) lower molar proportions data. Table S6. Strepsirrhine (n=1) lower molar proportions summary data. Table S7. Primate molar crown formation time data. Figure S1. Molar area correction coefficients method. Figure S2. Molar area correction coefficients test. Figure S3. Anthropoid lower molar proportions of individual specimens (n=2,895). Figure S4. Posterior density of molar proportion slope (interspecific). Figure S5. Posterior density of molar proportion intercept. Figure S6. Posterior density of relative M 2 area slope (interspecific). Figure S7. Posterior density of relative M 2 area intercept. Figure S8. Posterior density of proportion mediated (interspecific). Figure S9. Ln body mass as a function of fit/deviation from the dic molar proportion model line. Figure S10. Anthropoid lower molar proportions intra-specific variation. Figure S11. Anthropoid lower molar proportions with respect to relative M 2 area. Figure S12. Molecular phylogeny of anthropoid primates (n=100). Figure S13. Anthropoid lower molar proportions intra-specific error. Supplementary references. (1–43). (PDF 3891 kb). [file 12862_2016_673_MOESM1_ESM.pdf]

SUPPLEMENTARY INFORMATION

The evolution of anthropoid molar proportions

Katherine E Carter[1*†] and Steven Worthington[2†]

*Correspondence:
carter@fas.harvard.edu,
sworthington@iq.harvard.edu
[1]Department of Human Evolutionary Biology, Harvard University, 11 Divinity Avenue, 02138 Cambridge, USA
Full list of author information is available at the end of the article
†Equal contributor

Supplementary Tables

Table S1 Anthropoid ($n = 100$) lower molar proportions, body mass, and diet summary data

Genus	Species	n	M_1 area	M_2 area	M_3 area	M_2/M_1	M_3/M_1	BM*	PDC*
Alouatta	*belzebul*	13	35.41	41.24	41.12	1.17	1.16	6.39	fruit
Alouatta	*caraya*	20	32.62	38.91	41.70	1.19	1.28	5.38	leaves
Alouatta	*guariba*	18	34.04	39.87	42.60	1.17	1.25	5.54	leaves
Alouatta	*palliata*	18	39.23	45.86	43.78	1.17	1.12	6.25	leaves
Alouatta	*seniculus*	34	39.36	45.11	44.69	1.15	1.14	5.92	leaves
Ateles	*geoffroyi*	25	25.14	25.10	20.76	1.00	0.83	7.54	fruit
Ateles	*paniscus*	22	25.17	24.47	19.94	0.97	0.79	8.78	fruit
Brachyteles	*arachnoides*	8	42.40	42.40	34.08	1.00	0.81	8.84	leaves
Lagothrix	*lagotricha*	19	27.37	30.81	25.57	1.13	0.93	7.15	fruit
Callithrix	*jacchus*	13	4.95	3.35	0.00	0.67	0.00	0.37	omnivore
Callithrix	*pygmaea*	33	2.37	1.81	0.00	0.77	0.00	0.12	insects
Leontopithecus	*rosalia*	8	8.29	6.45	0.00	0.78	0.00	0.64	insects
Saguinus	*fuscicollis*	33	4.69	3.41	0.00	0.73	0.00	0.35	fruit
Saguinus	*midas*	55	4.74	3.30	0.00	0.70	0.00	0.55	insects
Saguinus	*oedipus*	36	5.77	3.79	0.00	0.66	0.00	0.41	insects
Aotus	*trivirgatus*	46	8.75	8.75	6.95	1.00	0.80	0.77	fruit
Cebus	*apella*	34	21.37	18.51	11.65	0.87	0.55	3.08	fruit
Cebus	*capucinus*	47	20.60	17.49	13.14	0.85	0.64	3.11	fruit
Saimiri	*oerstedii*	29	6.45	5.22	3.31	0.81	0.51	0.79	fruit
Saimiri	*sciureus*	39	6.76	5.53	3.53	0.82	0.52	0.82	animals
Cacajao	*calvus*	14	18.58	17.91	13.46	0.96	0.72	3.17	fruit
Callicebus	*moloch*	17	10.33	10.45	8.29	1.02	0.81	0.99	fruit
Callicebus	*torquatus*	20	11.00	10.73	7.89	0.98	0.72	1.25	fruit
Chiropotes	*satanas*	28	12.92	11.71	8.93	0.91	0.69	2.74	fruit
Pithecia	*pithecia*	23	11.59	11.94	10.99	1.03	0.95	1.76	fruit
Allenopithecus	*nigroviridis*	14	24.61	33.43	27.45	1.36	1.11	4.68	fruit
Allochrocebus	*lhoesti*	25	26.78	34.12	28.61	1.28	1.07	4.71	leaves
Allochrocebus	*preussi*	14	23.11	30.60	25.59	1.32	1.11	4.50	fruit
Cercopithecus	*ascanius*	50	18.51	24.94	20.54	1.35	1.11	3.31	fruit
Cercopithecus	*cephus*	40	20.17	26.37	20.99	1.31	1.04	3.58	fruit
Cercopithecus	*diana*	34	23.98	30.77	25.40	1.29	1.06	4.55	fruit
Cercopithecus	*erythrogaster*	6	20.55	27.57	21.23	1.34	1.03	3.25	fruit
Cercopithecus	*erythrotis*	9	19.54	25.43	20.71	1.30	1.06	3.25	fruit
Cercopithecus	*mitis*	37	25.20	33.61	27.71	1.33	1.10	4.89	fruit
Cercopithecus	*mona*	27	20.68	28.56	23.81	1.38	1.15	3.80	fruit
Cercopithecus	*neglectus*	37	24.15	33.12	28.68	1.38	1.19	5.70	fruit
Cercopithecus	*nictitans*	45	22.97	31.78	26.20	1.39	1.14	5.46	fruit
Cercopithecus	*petaurista*	17	20.87	27.97	22.63	1.34	1.09	4.13	leaves
Cercopithecus	*pogonias*	50	20.32	28.46	22.41	1.40	1.11	3.58	fruit
Cercopithecus	*wolfi*	36	18.57	26.19	21.93	1.41	1.18	3.39	fruit
Chlorocebus	*aethiops*	114	25.47	34.81	29.46	1.37	1.16	4.14	fruit
Erythrocebus	*patas*	29	31.92	45.09	38.07	1.42	1.20	3.87	omnivore
Miopithecus	*talapoin*	42	10.88	13.97	11.20	1.28	1.03	2.25	fruit
Colobus	*guereza*	60	38.07	48.66	56.86	1.28	1.49	8.26	leaves
Colobus	*polykomos*	84	35.67	43.82	49.75	1.23	1.40	9.10	leaves
Colobus	*satanas*	44	35.87	41.34	46.40	1.15	1.30	4.23	seeds
Nasalis	*larvatus*	15	36.90	48.76	58.86	1.32	1.60	15.11	leaves
Piliocolobus	*badius*	40	34.64	41.67	48.66	1.20	1.41	10.28	leaves
Piliocolobus	*kirkii*	11	26.22	31.40	36.80	1.20	1.40	5.63	leaves
Presbytis	*comata*	15	26.30	30.10	28.92	1.15	1.10	6.70	leaves
Presbytis	*melalophos*	30	24.19	26.99	23.78	1.12	0.98	6.53	fruit
Presbytis	*potenziani*	20	33.75	40.30	39.11	1.19	1.16	6.36	leaves
Presbytis	*rubicunda*	33	24.00	24.57	24.91	1.02	1.04	6.18	leaves
Presbytis	*thomasi*	7	27.11	30.14	31.37	1.11	1.16	6.73	leaves
Procolobus	*verus*	45	20.25	24.41	28.76	1.21	1.42	4.45	leaves
Pygathrix	*nemaeus*	21	32.71	40.99	48.55	1.26	1.49	9.72	leaves
Rhinopithecus	*roxellana*	5	44.41	62.02	72.92	1.40	1.65	14.75	leaves

Continued on next page . . .

Genus	Species	n	M_1 area	M_2 area	M_3 area	M_2/M_1	M_3/M_1	BM*	PDC*
Semnopithecus	*entellus*	100	42.05	54.40	62.26	1.29	1.47	10.21	leaves
Simias	*concolor*	15	29.99	39.33	46.98	1.31	1.57	7.97	leaves
Trachypithecus	*cristatus*	39	33.18	43.42	45.40	1.31	1.37	6.18	leaves
Trachypithecus	*francoisi*	8	30.72	36.24	40.63	1.18	1.32	7.88	leaves
Trachypithecus	*obscurus*	44	26.80	34.20	34.91	1.28	1.30	7.41	leaves
Trachypithecus	*pileatus*	13	34.35	42.09	43.61	1.23	1.27	11.34	leaves
Trachypithecus	*vetulus*	44	28.74	35.68	40.32	1.24	1.40	7.04	leaves
Cercocebus	*agilis*	21	41.03	56.50	51.47	1.38	1.26	7.38	omnivore
Cercocebus	*torquatus*	37	45.90	60.77	62.31	1.33	1.36	8.78	fruit
Lophocebus	*albigena*	69	32.88	44.70	40.85	1.36	1.25	6.57	fruit
Macaca	*fascicularis*	28	27.71	38.66	41.77	1.40	1.51	4.46	fruit
Macaca	*fuscata*	39	42.62	62.96	74.48	1.48	1.75	11.83	fruit
Macaca	*hecki*	24	38.92	52.71	55.11	1.36	1.42	9.00	fruit
Macaca	*maura*	7	37.37	55.38	66.30	1.49	1.78	7.88	fruit
Macaca	*mulatta*	61	34.04	49.00	58.21	1.44	1.71	6.61	fruit
Macaca	*nemestrina*	43	40.97	57.67	72.04	1.41	1.76	8.85	fruit
Macaca	*nigra*	40	36.34	52.78	60.63	1.46	1.67	7.68	fruit
Macaca	*ochreata*	9	35.78	48.82	57.88	1.36	1.62	3.95	fruit
Macaca	*silenus*	4	30.69	46.14	52.58	1.50	1.70	6.42	fruit
Macaca	*sinica*	39	28.18	38.71	41.16	1.37	1.46	4.44	fruit
Macaca	*sylvanus*	4	40.10	55.69	71.54	1.39	1.79	12.09	omnivore
Macaca	*tonkeana*	24	45.38	60.69	61.94	1.34	1.37	11.95	fruit
Mandrillus	*leucophaeus*	35	71.51	112.83	132.25	1.58	1.85	15.00	omnivore
Mandrillus	*sphinx*	10	66.50	108.27	124.72	1.65	1.89	23.60	omnivore
Papio	*anubis*	10	79.17	122.04	143.78	1.54	1.82	19.20	omnivore
Papio	*cynocephalus*	35	59.44	87.89	108.26	1.48	1.82	17.05	fruit
Papio	*hamadryas*	25	68.31	112.18	132.51	1.65	1.95	14.15	omnivore
Papio	*ursinus*	6	82.72	126.46	157.02	1.53	1.91	22.30	fruit
Theropithecus	*gelada*	25	66.05	103.18	132.48	1.56	2.01	15.35	leaves
Gorilla	*gorilla gorilla*	35	191.80	235.33	217.27	1.23	1.13	120.95	fruit
Homo	*sapiens*	20	107.16	108.92	109.52	1.02	1.02	67.10	omnivore
Pan	*paniscus*	25	79.32	85.83	70.11	1.08	0.89	39.10	fruit
Pan	*trog. schwein.*	26	101.22	114.49	101.23	1.13	1.00	38.20	fruit
Pan	*trog. trog.*	38	99.49	109.96	99.04	1.11	1.00	52.75	fruit
Pongo	*abelii*	7	131.40	154.23	143.31	1.17	1.09	56.75	fruit
Pongo	*pygmaeus*	38	140.16	157.27	144.31	1.12	1.03	57.15	fruit
Hoolock	*hoolock*	28	35.22	45.71	42.35	1.30	1.21	6.88	fruit
Hylobates	*agilis*	16	27.58	30.54	29.52	1.11	1.07	5.85	fruit
Hylobates	*klossii*	16	24.66	27.17	21.34	1.10	0.87	5.79	fruit
Hylobates	*lar*	32	28.63	33.76	32.61	1.18	1.14	5.64	fruit
Hylobates	*pileatus*	4	29.28	32.03	33.39	1.10	1.15	5.47	fruit
Nomascus	*concolor*	19	30.38	36.43	33.19	1.20	1.10	7.71	fruit
Symphalangus	*syndactylus*	26	44.42	55.00	51.40	1.24	1.16	11.24	fruit

*BM = body mass (kg), sources [1–7]. PDC = primary dietary category, sources: [8–35]

Table S2 Molar area proportion PGLMM posterior probabilities.

Taxon	Slope (Interspecific) $\mathbb{P} \in [1.95, 2.05]$	$\mathbb{P} \in [1.90, 2.10]$	$\mathbb{P} \in [1.85, 2.15]$
Anthropoidea	0.047	0.102	0.184
Platyrrhini	0.132	0.264	0.395
Platyrrhini*	0.082	0.168	0.259
Catarrhini	0.024	0.056	0.098
Hominoidea	0.001	0.003	0.005
Cercopithecoidea	0.075	0.162	0.253
Colobinae	0.015	0.034	0.054
Cercopithecinae	0.167	0.329	0.480
Cercopithecini	<0.001	<0.001	<0.001
Papionini	0.123	0.243	0.353
	Intercept		
Taxon	$\mathbb{P} \in [-0.95, -1.05]$	$\mathbb{P} \in [-0.90, -1.10]$	$\mathbb{P} \in [-0.85, -1.15]$
Anthropoidea	0.190	0.366	0.519
Platyrrhini	0.076	0.156	0.233
Platyrrhini*	0.186	0.352	0.502
Catarrhini	0.109	0.223	0.330
Hominoidea	0.006	0.011	0.018
Cercopithecoidea	0.139	0.272	0.408
Colobinae	0.022	0.040	0.059
Cercopithecinae	0.042	0.089	0.135
Cercopithecini	0.003	0.006	0.010
Papionini	0.053	0.109	0.164

$^{\mathbb{P}}$ posterior probability of parameter estimate being inside the ROPE
* non-callitrichin platyrrhines

Table S3 Relative M_2 area PGLMM posterior probabilities.

Taxon	Slope (Interspecific) $\mathbb{P} \in [0.328, 0.338]$	$\mathbb{P} \in [0.323, 0.343]$	$\mathbb{P} \in [0.318, 0.348]$
Anthropoidea	<0.001	<0.001	0.015
Platyrrhini	0.009	0.080	0.465
Platyrrhini*	0.009	0.061	0.270
Catarrhini	<0.001	0.001	0.044
Hominoidea	0.003	0.010	0.030
Cercopithecoidea	0.151	0.602	0.942
Colobinae	0.193	0.414	0.645
Cercopithecinae	0.245	0.678	0.941
Cercopithecini	<0.001	<0.001	<0.001
Papionini	0.259	0.554	0.811
	Intercept		
Taxon	$\mathbb{P} \in [-0.05, 0.05]$	$\mathbb{P} \in [-0.10, 0.10]$	$\mathbb{P} \in [-0.15, 0.15]$
Anthropoidea	0.028	0.054	0.080
Platyrrhini	0.062	0.125	0.198
Platyrrhini*	0.111	0.217	0.321
Catarrhini	0.024	0.047	0.071
Hominoidea	0.011	0.023	0.034
Cercopithecoidea	0.016	0.030	0.046
Colobinae	0.037	0.071	0.103
Cercopithecinae	<0.001	0.001	0.002
Cercopithecini	0.068	0.141	0.211
Papionini	0.010	0.018	0.028

$^{\mathbb{P}}$ posterior probability of parameter estimate being inside the ROPE
* non-callitrichin platyrrhines

Table S4 Proportion mediated (pr_m) PGLMM posterior probabilities.

	pr_m (Interspecific)	
Taxon	$\mathbb{P} \in [0.9, 1]$	$\mathbb{P} \in [0.8, 1]$
Anthropoidea	<0.001	0.009
Platyrrhini	<0.001	0.004
Platyrrhini*	0.003	0.014
Catarrhini	0.001	0.049
Hominoidea	0.141	0.294
Cercopithecoidea	0.233	0.802
Colobinae	0.274	0.607
Cercopithecinae	0.190	0.667
Cercopithecini	0.415	0.796
Papionini	0.262	0.652

$^{\mathbb{P}}$ posterior probability of parameter estimate being inside the ROPE
* non-callitrichin platyrrhines

Table S5 Modern human ($n = 66$) lower molar proportions.*

Population	M_2/M_1	M_3/M_1	Population	M_2/M_1	M_3/M_1
Australia	0.88	0.85	USA (Ohio)	0.92	0.95
Australia	1.05	1.10	USA (Ohio)	0.90	0.81
Australia	1.17	1.04	USA (Ohio)	0.94	0.90
Australia	0.94	0.79	USA (Ohio)	0.87	0.89
Australia	1.01	0.77	USA (Ohio)	0.83	0.94
Egypt	1.00	0.00	USA (Ohio)	0.93	0.88
Egypt	0.86	0.00	USA (Ohio)	0.91	0.82
Egypt	1.01	0.00	USA (Ohio)	0.84	0.97
Egypt	0.89	0.95	USA (Ohio)	0.79	0.73
Egypt	1.04	0.92	USA (Ohio)	0.85	0.71
Egypt	1.03	0.88	USA (Ohio)	0.87	0.71
Egypt	1.00	0.97	USA (Ohio)	1.02	0.78
Egypt	0.90	0.88	USA (Ohio)	0.85	0.78
Egypt	0.87	1.00	Yugoslavia	0.95	0.93
Egypt	0.89	0.88	Yugoslavia	1.07	0.95
Egypt	0.94	0.93	Yugoslavia	0.96	0.98
Egypt	0.95	0.00	Yugoslavia	0.84	0.91
Egypt	0.94	0.91	Yugoslavia	1.00	1.02
Egypt	0.92	0.91	Yugoslavia	0.93	0.79
Egypt	0.90	0.80	Yugoslavia	0.96	0.87
Egypt	1.00	0.86	Yugoslavia	0.91	0.84
Egypt	0.94	0.87	Yugoslavia	0.93	0.80
Egypt	0.95	0.97	Yugoslavia	0.86	0.89
Egypt	1.00	0.88	Yugoslavia	0.89	0.00
Egypt	1.05	0.91	Yugoslavia	0.95	0.81
Iceland	0.96	0.98	Yugoslavia	0.96	0.92
USA (Ohio)	0.93	0.77	Yugoslavia	0.81	0.00
USA (Ohio)	0.86	0.95	Yugoslavia	0.96	0.90
USA (Ohio)	0.89	0.82	Yugoslavia	0.81	0.88
USA (Ohio)	0.99	0.81	Yugoslavia	0.98	0.91
USA (Ohio)	0.85	0.92	Yugoslavia	1.08	0.90
USA (Ohio)	0.81	0.73	Yugoslavia	0.94	0.82
USA (Ohio)	0.93	0.82	Yugoslavia	1.02	0.89

* Data collected on specimens from the Museum of Comparative Zoology, Harvard.

Table S6 Strepsirrhine $(n = 1)$ lower molar proportions summary data

Genus	Species	n	M_1 area	M_2 area	M_3 area	M_2/M_1	M_3/M_1
Varecia	*variegata*	4	33.65	30.09	16.82	0.89	0.50

source: [36].

Table S7 Primate molar crown formation time data

Genus	Species	M_1Et*	M_2Et	M_3Et	Overlap*
Macaca	*fascicularis*	1.38	3.38	5.50	−0.11
Macaca	*mulatta*	1.35	3.15	5.60	−0.10
Papio	*cynocephalus*	1.64	3.79	6.21	0.37
Gorilla	*gorilla*	3.50	6.58	10.38	0.29
Homo	*sapiens*	6.23	11.75	20.10	−0.00
Pan	*troglodytes*	3.26	6.46	10.50	0.17
Pongo	*pygmaeus*	3.50	5.00	10.00	0.22
Hylobates	*lar*	1.90	2.88	6.70	0.12
Varecia	*variegata*	0.48	0.69	1.13	0.38

* Et = eruption time, Overlap = temporal overlap,
sources: [37–42].

Supplementary Figures

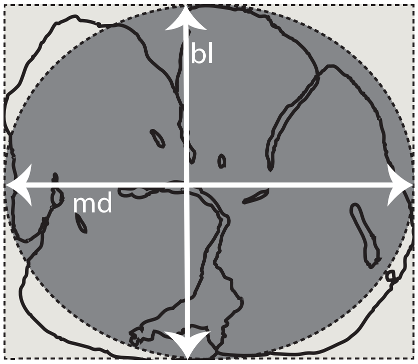

Figure S1 Molar area correction coefficients method. Illustration of the method for deriving molar area correction coefficients. Teeth of known area are inscribed in a rectangle ra (outer dashed line) and an ellipse ea (inner dashed line), then the coefficient x best fitting the data is derived using the formula: $ca = ra\,(x) + ea\,(1 - x)$.

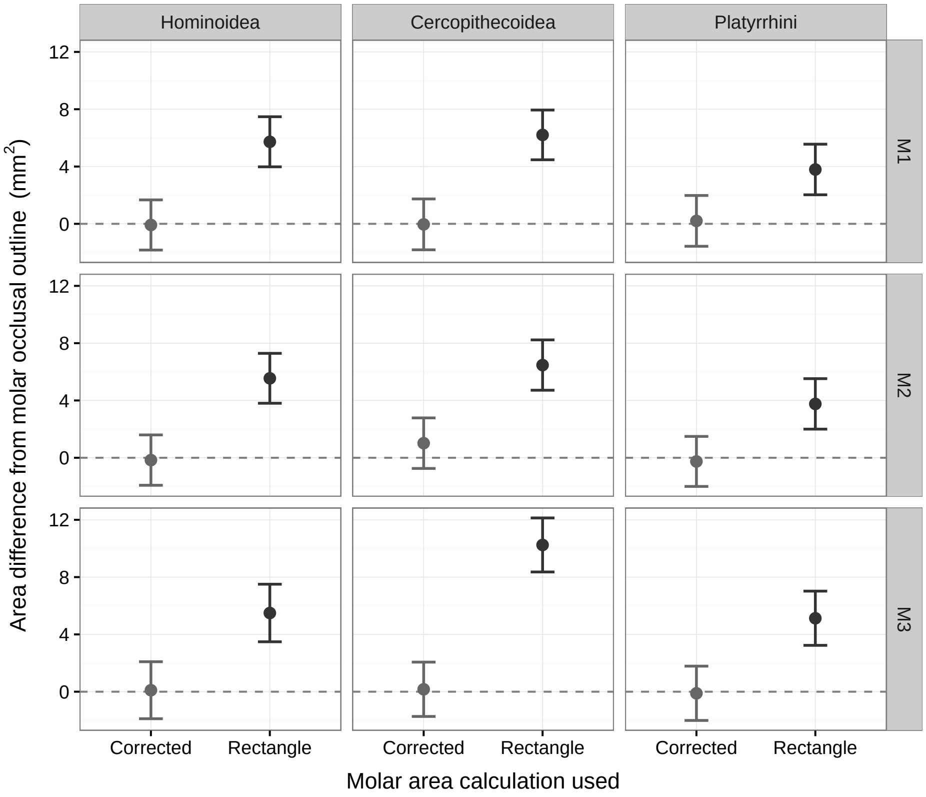

Figure S2 Molar area correction coefficients test. Differences in molar area (mm^2) between those calculated by tracing polygons around molar occlusal outlines on photographs and those calculated: 1) as the product of linear length and breadth measurements ('rectangle'), and 2) using the correction coefficient method outline in the main article methods section ('corrected'). Points and error bars are mean estimates and 95% confidence intervals, respectively, from a mixed effects regression model. Comparisons are broken down by taxon and molar type.

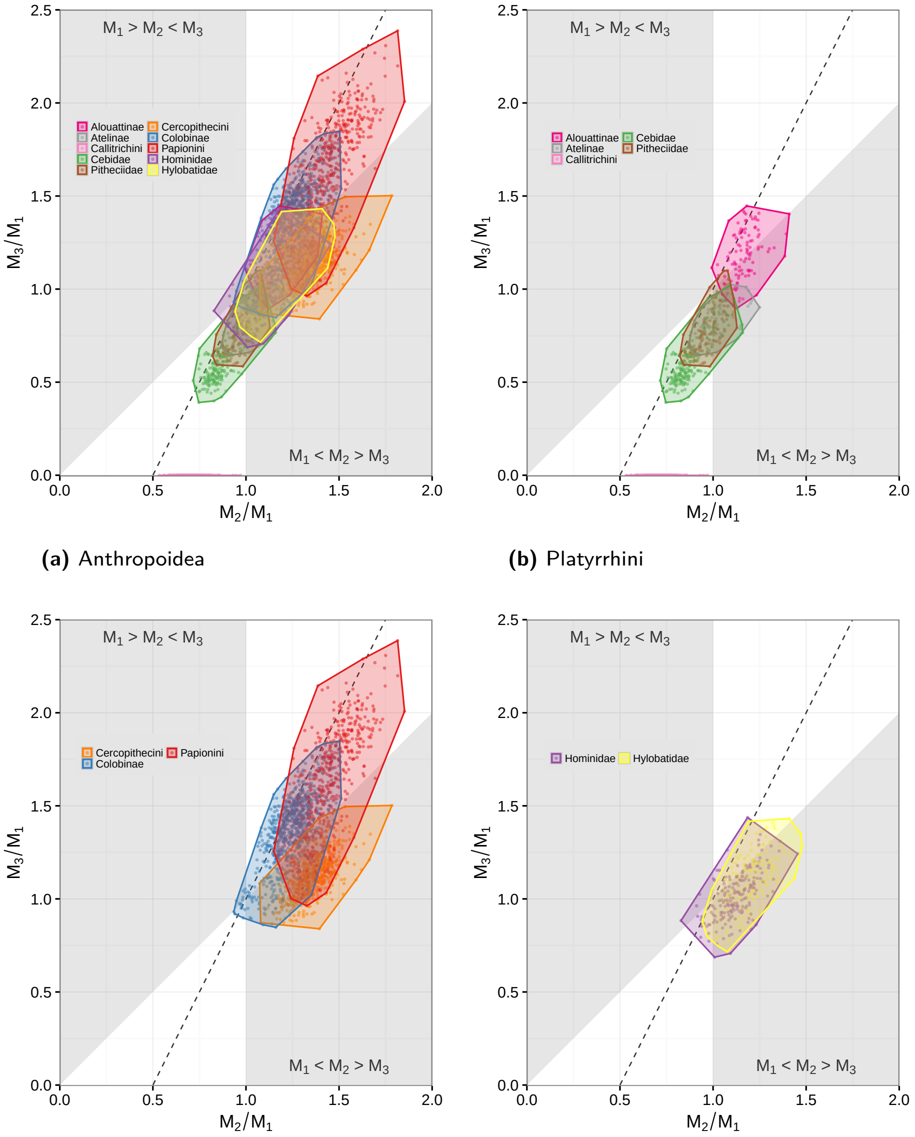

(a) Anthropoidea **(b)** Platyrrhini

(c) Cercopithecoidea **(d)** Hominoidea

Figure S3 Anthropoid lower molar proportions of individual specimens (n=2,895). Lower molar proportions for 2,895 specimens of 100 anthropoid species. Points are individual specimens. Convex hulls denote the range of species values for each higher ranked taxon. Dashed line indicates DIC model's predicted relationship between molar proportion ratios: $M_3/M_1 = 2(M_2/M_1) - 1$. White regions are locations in molar proportion morphospace consistent with the DIC model: a high a/i region where $M_1 < M_2 < M_3$ and a low a/i region where $M_1 > M_2 > M_3$.

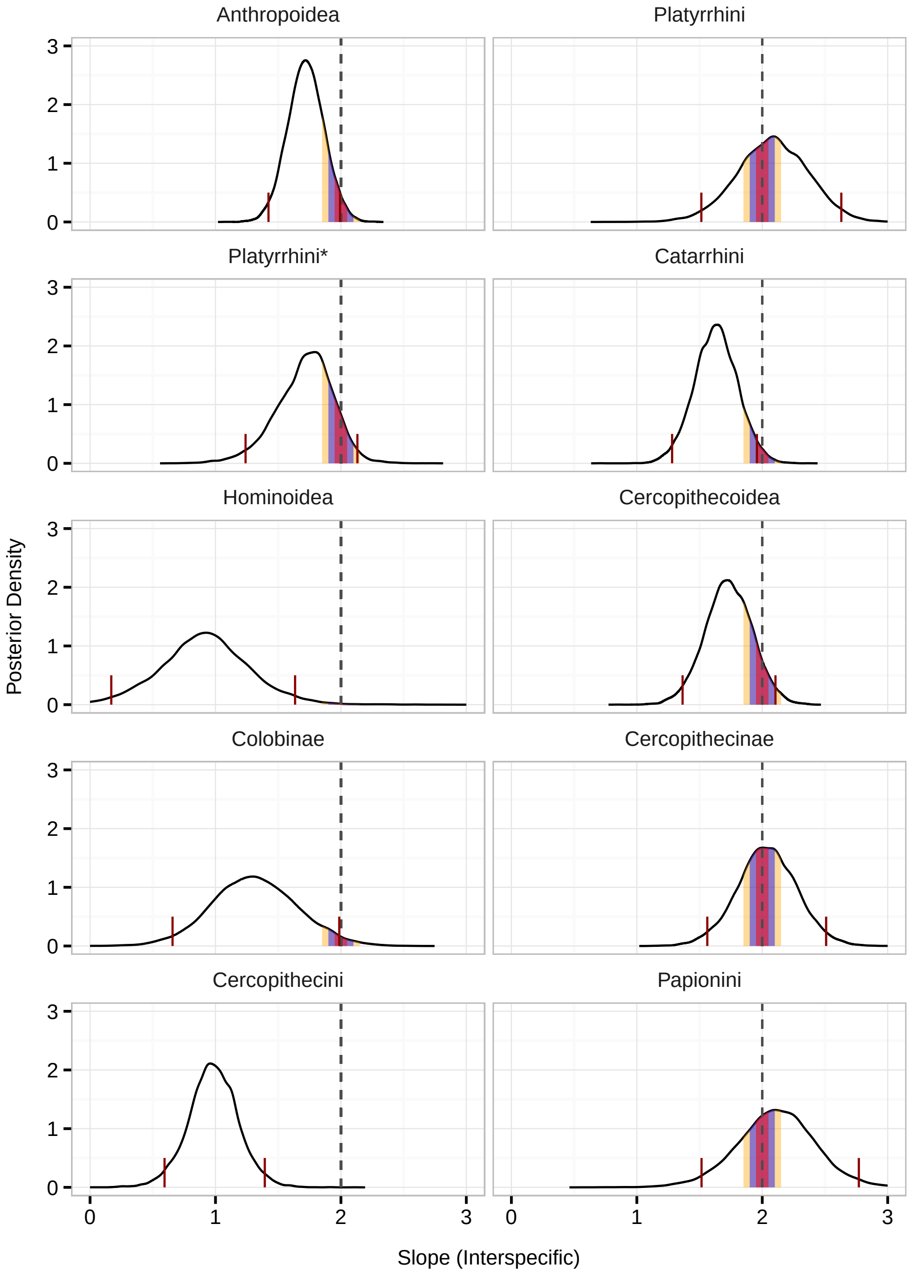

Figure S4 Posterior density of molar area proportion slope (interspecific). Black solid line depicts posterior density of molar area proportion interspecific slope. Red solid lines depict 95% HDI. Black dashed line depicts DIC model expectation. Colored polygons within posterior density depict different ROPE sizes: red = $\mathbb{P} \in [1.95, 2.05]$, blue = $\mathbb{P} \in [1.90, 2.10]$, orange = $\mathbb{P} \in [1.85, 2.15]$. *non-callitrichin platyrrhines.

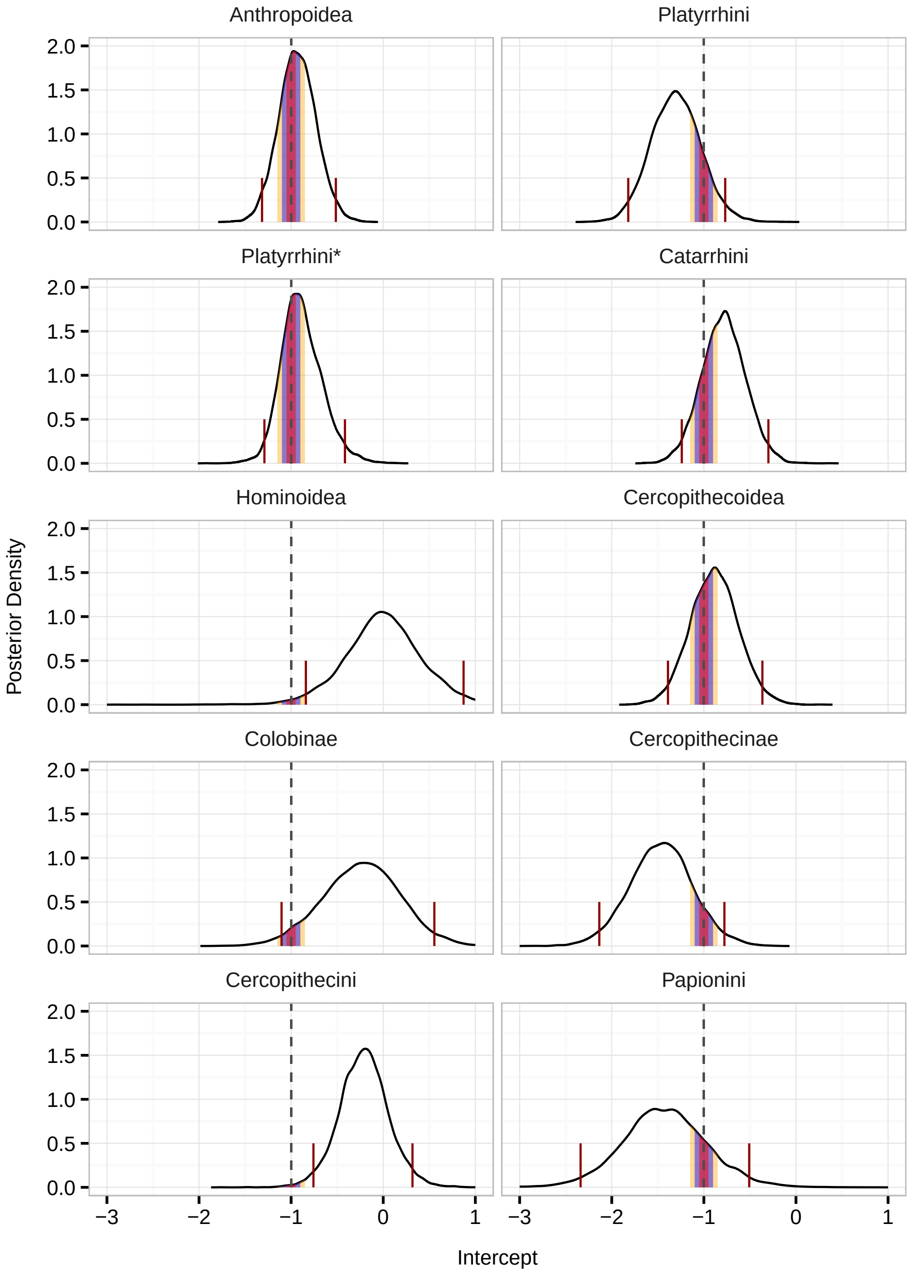

Figure S5 Posterior density of molar area proportion intercept. Black solid line depicts posterior density of molar area proportion intercept. Red solid lines depict 95% HDI. Black dashed line depicts DIC model expectation. Colored polygons within posterior density depict different ROPE sizes: red $= \mathbb{P} \in [-0.95, -1.05]$, blue $= \mathbb{P} \in [-0.90, -1.10]$, orange $= \mathbb{P} \in [-0.85, -1.15]$. *non-callitrichin platyrrhines.

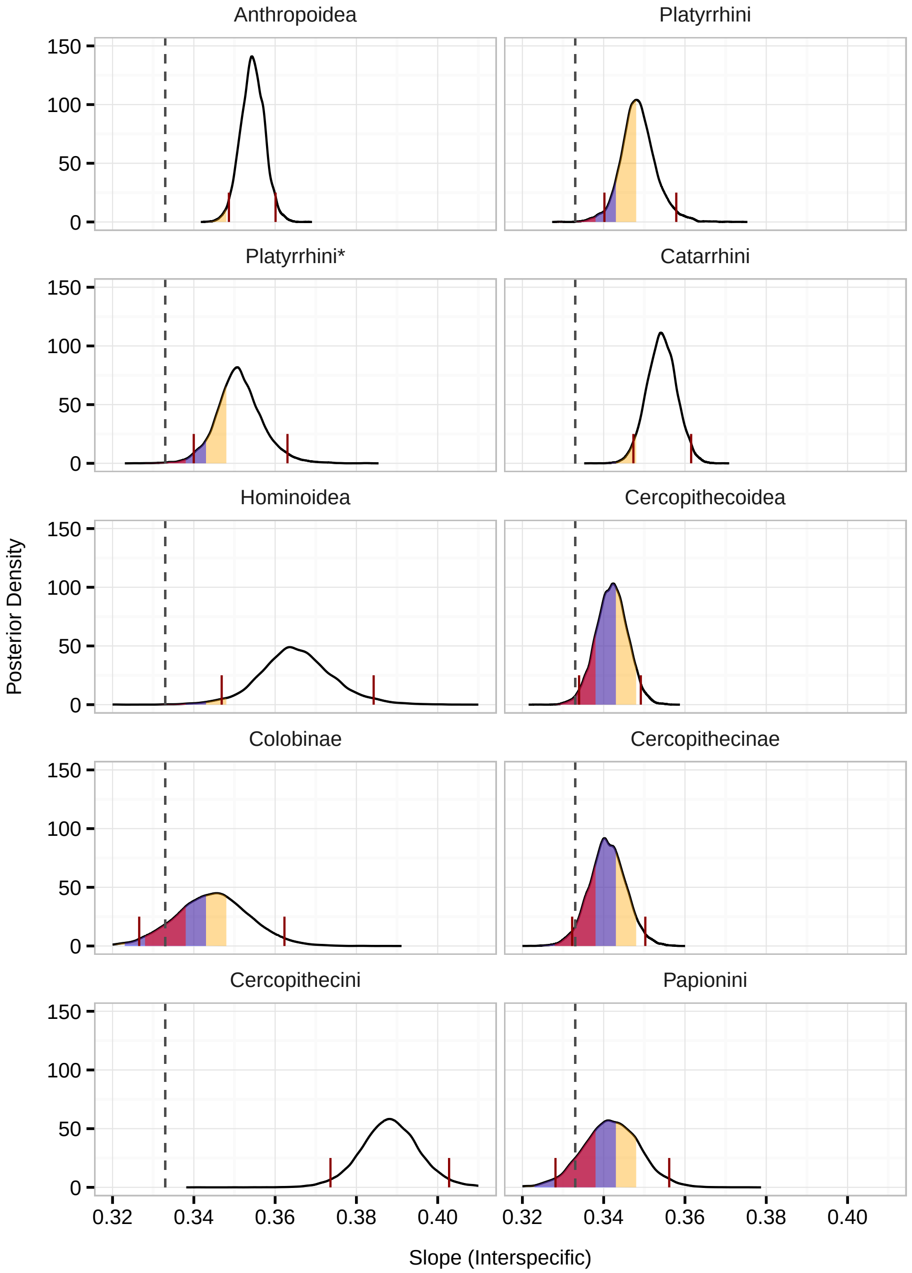

Figure S6 Posterior density of relative M_2 area slope (interspecific). Black solid line depicts posterior density of relative M_2 area interspecific slope. Red solid lines depict 95% HDI. Black dashed line depicts DIC model expectation. Colored polygons within posterior density depict different ROPE sizes: red $= \mathbb{P} \in [0.328, 0.338]$, blue $= \mathbb{P} \in [0.323, 0.343]$, orange $= \mathbb{P} \in [0.318, 0.348]$. *non-callitrichin platyrrhines.

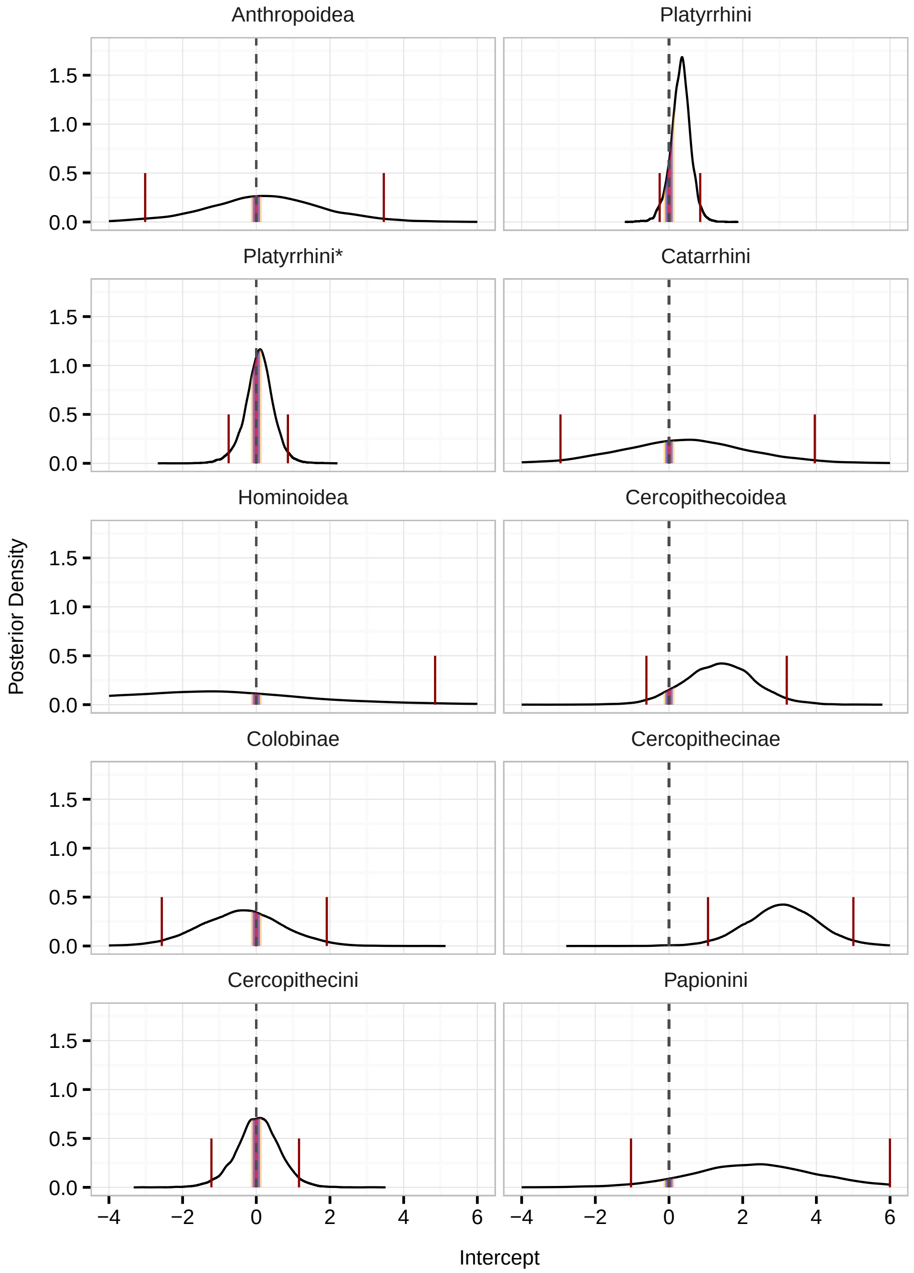

Figure S7 Posterior density of relative M_2 area intercept. Black solid line depicts posterior density of relative M_2 area intercept. Red solid lines depict 95% HDI. Black dashed line depicts DIC model expectation. Colored polygons within posterior density depict different ROPE sizes: red $= \mathbb{P} \in [-0.05, 0.05]$, blue $= \mathbb{P} \in [-0.10, 0.10]$, orange $= \mathbb{P} \in [-0.15, 0.15]$. *non-callitrichin platyrrhines.

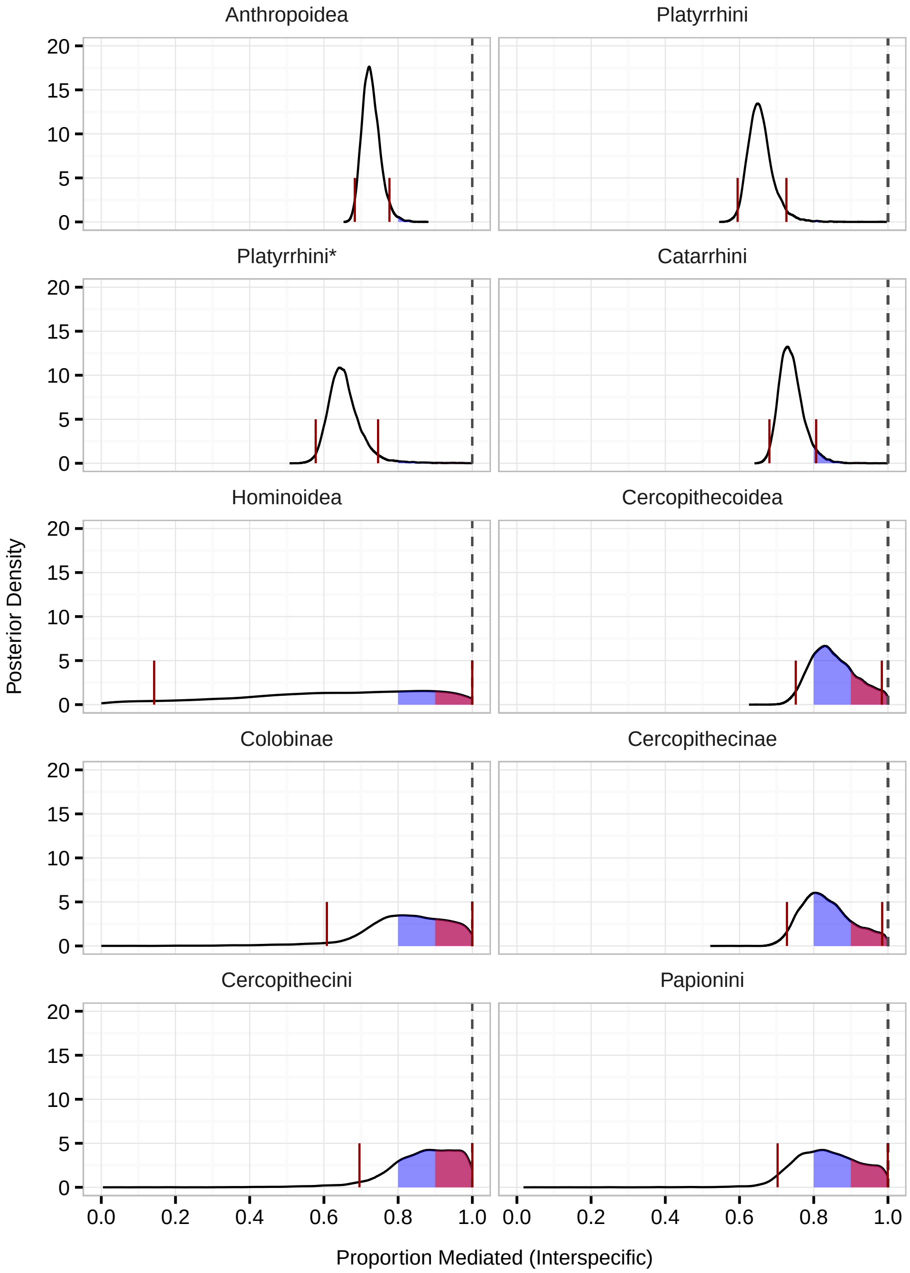

Figure S8 Posterior density of proportion mediated (interspecific). Black solid line depicts posterior density of proportion mediated (interspecific). Red solid lines depict 95% HDI. Black dashed line depicts DIC model expectation. Colored polygons within posterior density depict different ROPE sizes: red $= \mathbb{P} \in [0.9, 1]$, blue $= \mathbb{P} \in [0.8, 1]$. *non-callitrichin platyrrhines.

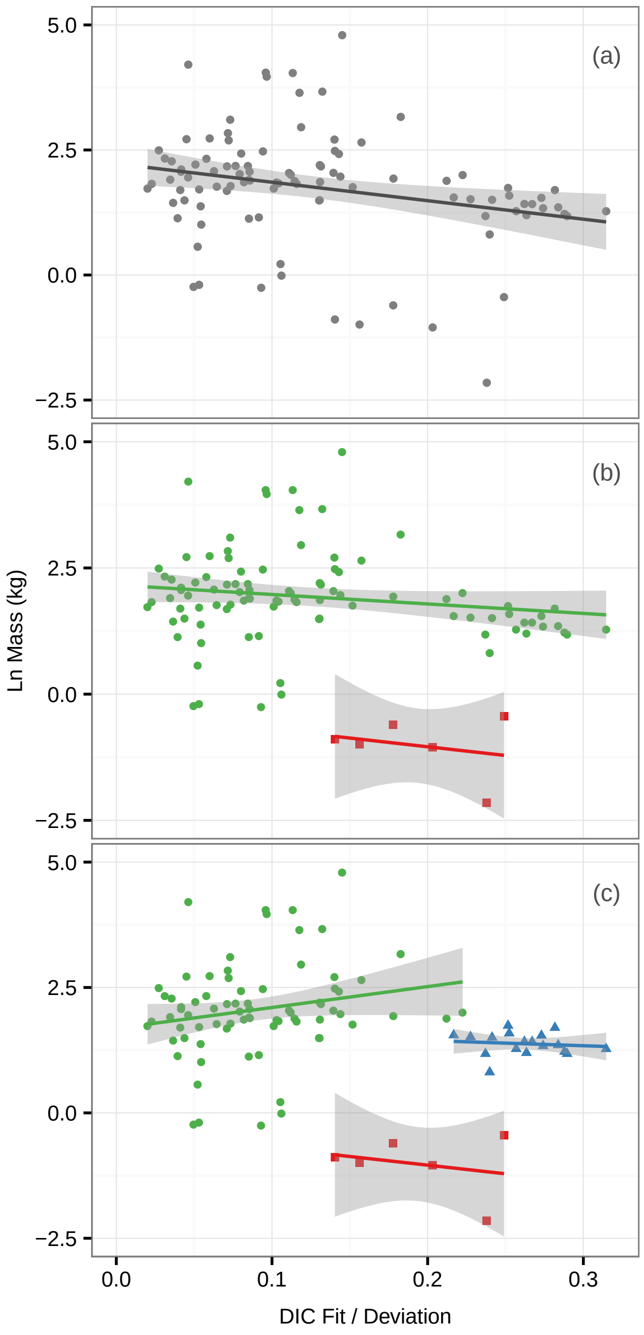

Figure S9 Ln body mass as a function of fit/deviation from the DIC molar proportion model line. Species mean ln body mass as a function of absolute perpendicular fit/deviation from the DIC model line. (a) Pooled regression for all anthropoid ($n = 100$) species. (b) Separate regression slopes and intercepts for callitrichins (red squares) and other anthropoids (green circles). (c) Separate regression slopes and intercepts for callitrichins (red squares), cercopithecins (blue triangles), and other anthropoids (green circles).

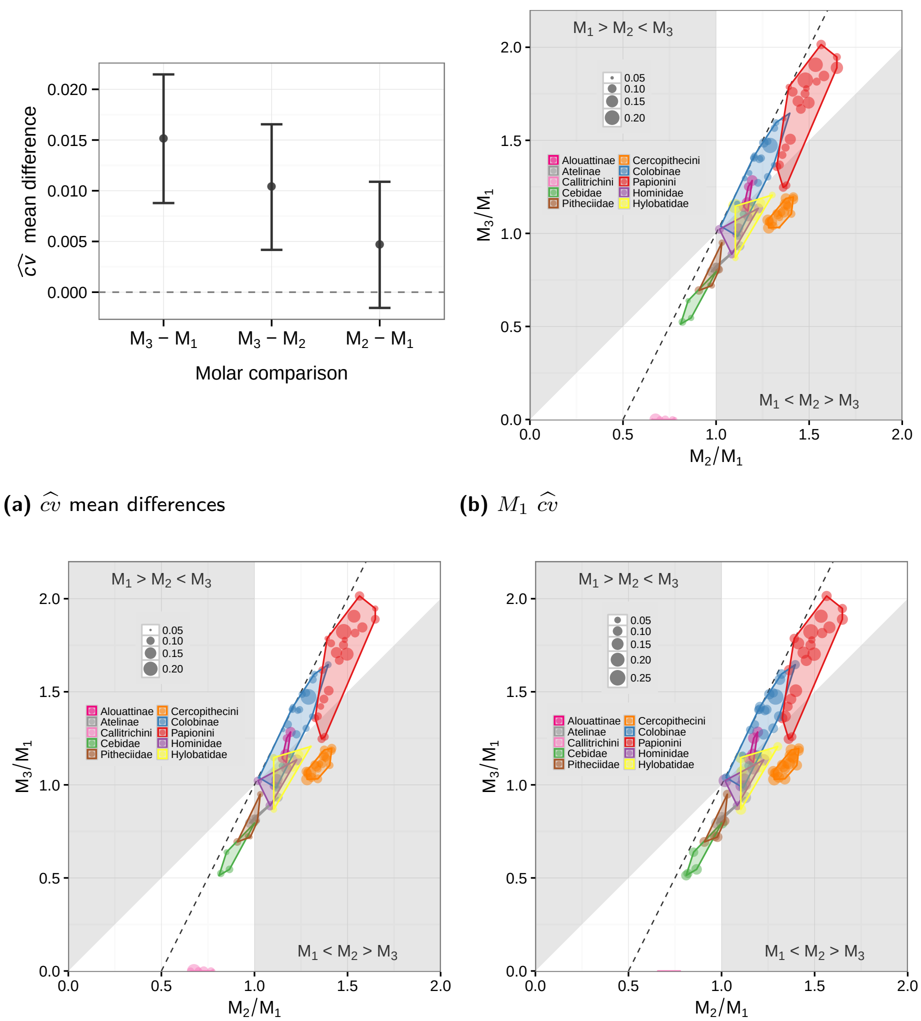

(a) $\widehat{cv}$ mean differences

(b) M_1 $\widehat{cv}$

(c) M_2 $\widehat{cv}$

(d) M_3 $\widehat{cv}$

Figure S10 Anthropoid lower molar proportions intra-specific variation. (a) mean differences and 95% HDIs of the coefficient of variation for small sample sizes ($\widehat{cv}$) for each lower molar type comparison. (b-d) Lower molar proportions for 100 anthropoid primates. Points are species mean values. Radii of points are scaled by $\widehat{cv}$ for M_{1-3}. Convex hulls denote the range of species values for each higher ranked taxon. Dashed line indicates DIC model's predicted relationship between molar proportion ratios: $M_3/M_1 = 2(M_2/M_1) - 1$. White regions are locations in molar proportion morphospace consistent with the DIC model: a high a/i region where $M_1 < M_2 < M_3$ and a low a/i region where $M_1 > M_2 > M_3$.

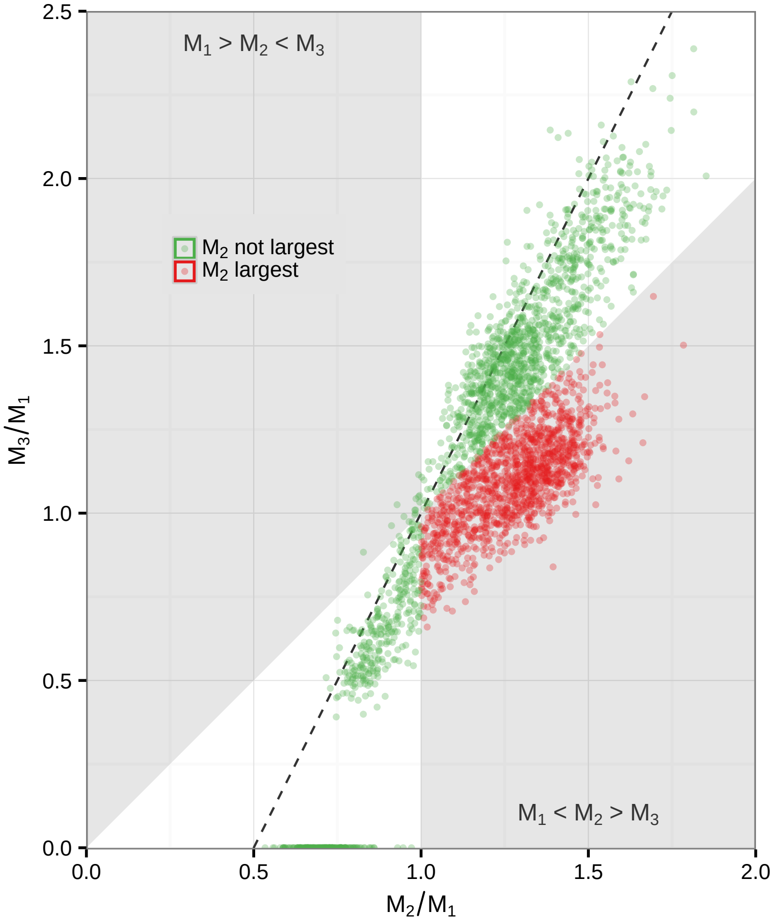

Figure S11 Anthropoid lower molar proportions with respect to relative M_2 area. Lower molar proportions of individual specimens ($n = 2,895$) from 100 anthropoid primates. Observations have been dichotomized according to whether they fall in regions of the morphospace where M_2 is, or is not, the largest lower molar.

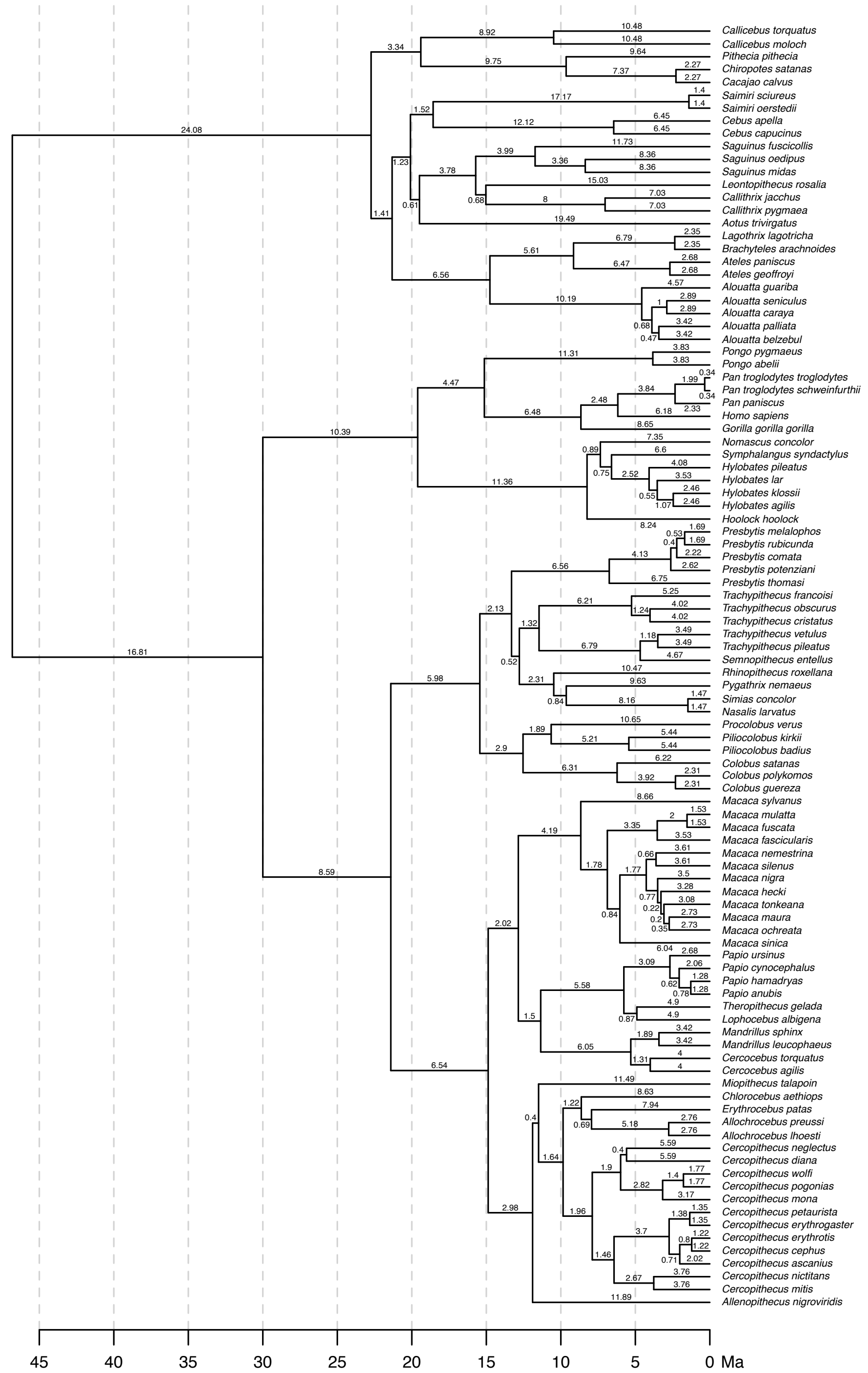

Figure S12 Molecular phylogeny of anthropoid primates ($n = 100$). Molecular-based consensus phylogeny for 100 anthropoid primates. Topology and branch lengths were extracted from version 3 of the 10K Trees database of primate phylogenetic relationships [43].

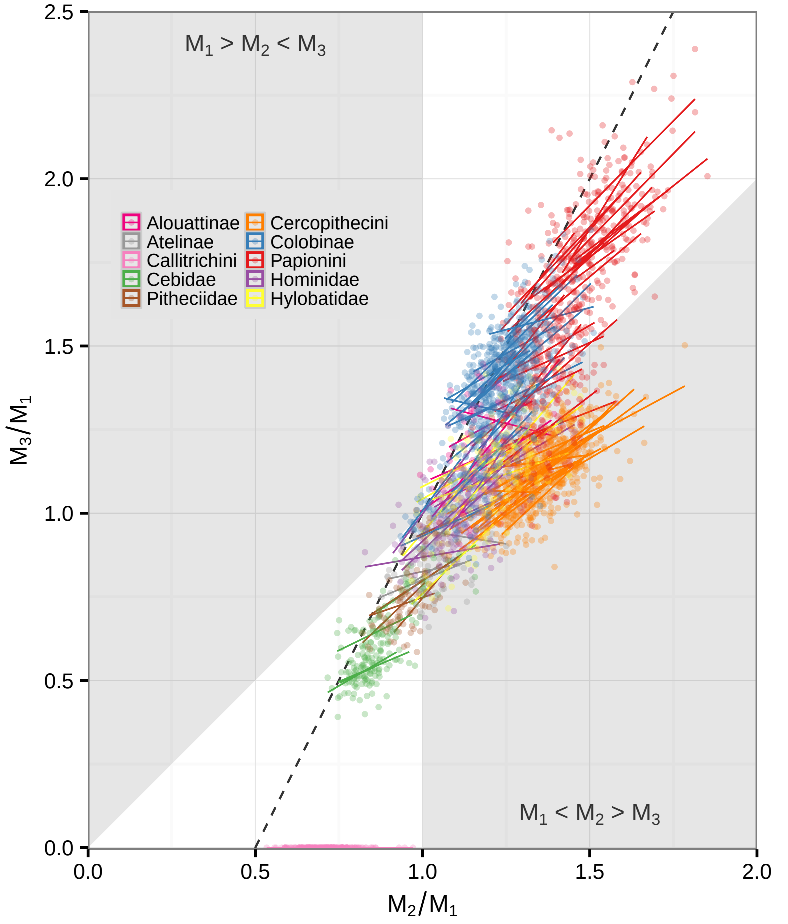

Figure S13 Anthropoid lower molar proportions intra-specific error. Lower molar proportions for 100 anthropoid primates. Points are species mean values. Lines are PGLMM estimates of slope and intercept for each species. Dashed line indicates DIC model's predicted relationship between molar proportion ratios: $M_3/M_1 = 2(M_2/M_1) - 1$. White regions are locations in molar proportion morphospace consistent with the DIC model: a high a/i region where $M_1 < M_2 < M_3$ and a low a/i region where $M_1 > M_2 > M_3$.

Author details
[1]Department of Human Evolutionary Biology, Harvard University, 11 Divinity Avenue, 02138 Cambridge, USA.
[2]Institute for Quantitative Social Science, Harvard University, 1737 Cambridge Street, 02138 Cambridge, USA.

References

1. Colyn, M.: Données pondérales sur les primates Cercopithecidae d'Afrique Centrale (Bassin du Zaïre/Congo). Mammamlia **58**, 483–487 (1994)
2. Delson, E., Terranova, C.J., Jungers, W.L., Sargis, E.J., Jablonski, N.G., Dechow, P.C.: Body mass in Cercopithecidae (Primates, Mammalia): estimation and scaling in extinct and extant taxa. Am Mus Nat Hist, Anthropol Papers **83**, 1–159 (2000)
3. Geissmann, T.: Evolution of Communication in Gibbons (Hylobatidae). Ph.D. Dissertation. PhD thesis, University of Zürich, Switzerland (1993)
4. Gordon, A.D.: Scaling of size and dimorphism in Primates II: macroevolution. Int J Primatol **27**(1), 63–105 (2006)
5. Holloway, R.L.: Within-species brain-body weight variability: a reexamination of the Danish data and other primate species). Am J Phys Anthropol **53**, 109–121 (1980)
6. Peres, C.A.: Which are the largest New World monkeys? J Hum Evol **26**, 245–249 (1994)
7. Smith, R.J., Jungers, W.L.: Body mass in comparative primatology. J Hum Evol **32**, 523–559 (1997)
8. Bartlett, T.Q.: The hylobatidae: small apes of asia. In: Campbell, C.J., Fuentes, A., MacKinnin, K.C., Panger, M., Bearder, S.K. (eds.) Primates in Perspective, pp. 274–289. Oxford University Press, New York (2007)
9. Beeson, M., Tame, S., Keeming, E., Lea, S.E.G.: Food habits of guenons (*Cercopithecus* spp.) in Afro-montane forest. Afr J Ecol **34**, 202–210 (1996)
10. Bowler, M., Bodmer, R.E.: Diet and food choice in Peruvian red uakaris (*Cacajao calvus ucayalii*): selective or opportunistic seed predation? Int J Primatol **32**, 1109–1120 (2011)
11. Buzzard, P.J.: Ecological partitioning of *Cercopithecus campbelli*, *C. petaurista* and *C. diana* in the Taï Forest. Int J Primatol **27**, 529–558 (2006)
12. Chapman, C.A., Chapman, L.J., Cords, M., Gathua, J.M., Gautier-Hion, A., Lambert, J.E., Rode, K., Tutin, C.E.G., White, L.J.T.: Variation in the diets of emphCercopithecus species: differences within forests, among forests, and across species. In: Glenn, M.E., Cords, M. (eds.) The Guenons: Diversity and Adaptation in African Monkeys, pp. 325–350. Kluwer Academic/Plenum Publishers, New York (2002)
13. Chiarello, A.G.: Diet of the brown howler monkey *Alouatta fusca* in a semi-deciduous forest fragment of Southeastern Brazil. Primates **35**(1), 25–34 (1994)
14. Chivers, D.: The Siamang in Malaya: A Field Study of a Primate in Tropical Rain Forest. Karger, Basel (1974)
15. de Castro, C.S.S., Araujo: Diet and feeding behavior of marmoset, *Callithrix Jacchus*. Rev Brasil Ecol **7**, 14–19 (1996)
16. Enstam, K.L., Isbell, L.A.: The guenons (genus *Cercopithecus*) and their allies. In: Campbell, C.J., Fuentes, A., MacKinnin, K.C., Panger, M., Bearder, S.K. (eds.) Primates in Perspective, pp. 252–274. Oxford University Press, New York (2007)
17. Kane, E.E., Bitty, E.A., McGraw, W.S.: Influence of association with red colobus (*Procolobus badius*) on the feeding ecology of Diana monkeys (*Cercopithecus diana*) in the Ivory Coast's Taï Forest. Am J Phys Anthropol **S54**, 177–177 (2012)
18. Kaplin, B.: Ranging behaviors of two species of guenons (*Cercopithecus lhoesti* and *C. mitis doggetti*) in the Nyungwe forest reserve, Rwanda. International Journal of Primatology **22**(4), 521–548 (2001)
19. Koenig, A., Borries, C.: Socioecology of Hanuman langurs: the story of their success. Evol Anthropol **10**, 122–137 (2001)
20. Pavelka, M.S.M., Knopff, K.H.: Diet and activity in black howler monkeys (*Alouatta pigra*) in southern Belize: does degree of frugivory influence activity level? Primates **45**, 105–111 (2004)
21. Peres, C.A.: Diet and feeding ecology of gray woolly monkeys (*Lagothrix lagothricha cana*) in Central Amazonia: comparisons with other atelines. Int J Primatol **15**(3), 333–372 (1994)
22. Pinto, L.P., Setz, E.Z.: Diet of *Alouatta belzebul discolor* in an Amazonian rain forest of Northern Mato Grosso State, Brazil. Int J Primatol **25**(6), 1197–1211 (2004)
23. Porter, L.M.: Dietary differences among sympatric callitrichines in northern Bolivia: *Callimico goeldii*, *Saguinus fuscicollis* and *Saguinus labiatus*. Int J Primatol **22**(6), 961–992 (2001)
24. Prates, H.M., Bicca-Marques, J.C.: Age-sex analysis of activity budget, diet, and positional behavior in *Alouatta caraya* in an orchard forest. Int J Primatol **29**, 703–715 (2008)
25. Preece, G.: Factors influencing variation in the population densities of *Colobus guereza*, within selectively logged forest at the Budongo Forest Reserve. In: Newton Fisher, N.E., Norman, H., Paterson, J.D., Reynolds, V. (eds.) Primates of Western Uganda, pp. 23–43. Springer, New York (2006)
26. Priston, N.E.C.: Crop-raiding by *Macaca ochreata brunnescens* in sulawesi: Reality, perceptions and outcomes for conservation. PhD thesis, University of Cambridge, Cambridge (2005)
27. Quyet, L.K., Duc, N.A., Tai, V.A., Wright, B.W., Covert, H.H.: Diet of the Tonkin snub-nosed monkey (*Rhinopithecus avuncular*) in the Khau Ca area, Ha Giang Province, Northeastern Vietnam. Vietnamese J Primatol **1**, 75–83 (2007)
28. Raemaekers, J.J.: Ecology of sympatric gibbons. Folia Primatol **31**, 227–245 (1979)
29. Robinson, J.G., Redford, K.H.: Body size, diet, and population density of Neotropical forest mammals. Am Nat **128**, 665–680 (1986)
30. Rosenberger, A.L.: Evolution of feeding niches in New World monkeys. Am J Phys Anthropol **88**, 525–562 (1992)
31. Sarmiento, E.E., Kingdon, J.: *Cercopithecus denti* Dent's Monkey. In: Butynski, T.M., Kingdon, J., Kalina, J. (eds.) Mammals of Africa. Volume II: Primates, pp. 330–333. Bloomsbury Publishing, London (2013)
32. Silver, S.C., Ostro, L.E.T., Yeager, C.P., Horwich, R.: Feeding ecology of the black howler monkey (*Alouatta pigra*) in Northern Belize. Am J Primatol **45**, 263–279 (1998)

33. Tsuji, Y., Fujita, S., Sugiura, H., Saito, C., Takatsuki, S.: Long-term variation in fruiting and the food habits of wild Japanese macaques on Kinkazan Island, northern Japan. Am J Primatol **68**, 1068–1080 (2006)
34. Zeeve, S.R.: Swamp monkeys of the Lomako Forest, Central Zaire. Primate Conservation **5**, 32–33 (1985)
35. Zhou, Q., Wei, F., Li, M., Huang, C., Luo, B.: Diet and food choice of (*Trachypithecus francoisi*) in the Nonggang Nature Reserve, China. Int J Primatol **27**(5), 1441–1460 (2006)
36. Swindler, D.R.: Primate Dentition: An Introduction to the Teeth of Non-human Primates. Cambridge University Press, Cambridge (2002)
37. Beynon, A.D., Dean, M.C., Reid, D.J.: Histological study on the chronology of the developing dentition in *Gorilla* and *Orangutan*. Am J Phys Anthropol **86**, 189–203 (1991)
38. Reid, D.J., Beynon, A.D., Ramirez Rozzi, F.V.: Histological reconstruction of dental development in four individuals from a medieval site in Picardie, France. J Hum Evol **35**, 463–477 (1998)
39. Schwartz, G.T., Mahoney, P., Godfrey, L.R., Cuozzo, F.P., Jungers, W.L., Randria, G.F.N.: Dental development in *Megaladapis edwardsi* (Primates, Lemuriformes): implications for understanding life history variation in subfossil lemurs. J Hum Evol **49**, 702–721 (2005)
40. Schwartz, G.T., Reid, D.J., Dean, M.C., Zihlman, A.L.: A faithful record of stressful life events preserved in the dental developmental record of a juvenile *Gorilla*. Int J Primatol **27**(4), 1201–1219 (2006)
41. Dirks, W., Bowman, J.E.: Life history theory and dental development in four species of catarrhine primates. J Hum Evol **53**, 309–320 (2007)
42. Smith, T.M., Reid, D.J., Dean, M.C., Olejniczak, A.J., Martin, L.B.: Molar development in common chimpanzees (*Pan troglodytes*). J Hum Evol **52**(2), 201–216 (2007)
43. Arnold, C., Matthews, L.J., Nunn, C.L.: The 10kTrees website: a new online resource for primate phylogeny. Evol Anthropol **19**(3), 114–118 (2010)
